# Digital Twin Brain: Generating Multitask Behavior from Connectomes for Personalized Therapy

**DOI:** 10.34133/bmef.0231

**Published:** 2026-02-12

**Authors:** Yuta Takahashi, Takafumi Soda, Hiroaki Tomita, Yuichi Yamashita

**Affiliations:** ^1^ Department of Information Medicine, National Center of Neurology and Psychiatry, Tokyo, Japan.; ^2^ Department of Psychiatry, Graduate School of Medicine, Tohoku University, Sendai, Japan.

## Abstract

**Objective:** This study introduces and validates a digital twin brain framework designed to translate an individual’s brain connectome into predictions of multitask neurobehavioral dynamics and personalized functional modulations. **Impact Statement:** We introduce a novel 2-component architecture—where a hypernetwork personalizes a main network from an individual’s connectome—establishing a mechanistic platform to simulate and design personalized interventions by directly linking connectomes to behavior. **Introduction:** Personalized psychiatry requires digital twin models that can predict functions across multiple domains, such as affective and cognitive processing, from an individual’s unique neurobiology. However, existing models struggle to bridge the gap between brain structure and complex, multitask behavior, limiting their clinical utility. **Methods:** A hypernetwork uses an individual’s resting-state connectome to generate parameters for a main recurrent neural network that simulates participant-specific behavioral and blood-oxygen-level-dependent (BOLD) time series across tasks. Leveraging the model’s end-to-end architecture linking connectomes to behavior, we used gradient backpropagation to identify connectome manipulations designed to selectively modulate affective or cognitive functions. **Results:** Validated on 228 individuals, the model predicted behavioral choices with over 90% accuracy, reaction times (*r* > 0.85), and BOLD patterns (*r* = 0.84) with high fidelity. Crucially, in silico interventions successfully modulated targeted functions and reproduced realistic, interindividual variability in treatment effects arising from each person’s baseline connectome. **Conclusion:** This digital twin brain system enables high-fidelity, in silico prediction and personalized modulation of complex neurobehavioral functions, advancing the potential for individualized psychiatric care.

## Introduction

The realization of personalized medicine is an important goal in modern medicine. Toward achieving this, the development of “digital twins”—mathematical models that mimic an individual’s organ functions to simulate and predict treatment responses [1]—is gaining substantial attention. In the context of brain modeling, approaches to constructing these digital replicas can be broadly categorized by their level of biological detail. While some digital twin approaches emphasize biophysical structure (e.g., neural mass models constrained by white matter tracts) [2,3], others aim to capture functional input–output relationships [1,4]. This technology holds potential as a powerful tool for advancing personalized medicine, particularly for psychiatric disorders, where the underlying mechanisms and symptom manifestations vary significantly among patients [5]. However, constructing a functional “digital twin brain” capable of capturing the complexity of psychiatric disorders faces several major challenges [4,6,7].

The first challenge is the gap between neurobiological substrates and higher-order cognition and behavior that define psychiatric symptoms [8,9]. Research aimed at bridging this gap has 2 main streams [9]. In the bottom-up tradition, biophysically grounded simulations are personalized using an individual’s connectome or microstructural data (e.g., neural mass and neural field models) [10,11]. These models can reproduce resting-state functional connectivity and even epileptic seizure dynamics, yet they face difficulties in modeling complex higher-order cognitive functions [2,9]. Conversely, the top-down tradition begins with abstract algorithms such as Bayesian inference, reinforcement learning, or active-inference agents [8,12–14]. Although these frameworks capture between-patient variability in cognition and behavior, they are usually fitted to behavioral data and only post hoc related to neural signals via correlation; the structural wiring and physiology of the individual play no causal role in their generative machinery [8]. Thus, neither existing approach has successfully linked neurobiological substrates with higher-order cognition in a dynamic manner. Therefore, the development of models bridging this divide is an urgent priority for a truly personalized, mechanistic digital twin brain.

The second challenge is the difficulty of unifying multiple functional domains affected by psychiatric disorders within a single model. Most psychiatric disorders involve impairments across several domains, not just one [8]. For instance, the National Institute of Mental Health’s Research Domain Criteria (RDoC) framework highlights the need to understand dysfunction across different functional domains [15]. For instance, in depression, individuals often exhibit deficits in both the Negative Valence Systems domain (e.g., altered affective responses) and the Cognitive Systems domain (e.g., reduced processing speed) [16,17]. However, most current brain modeling research focuses on single domains or tasks, failing to capture such complex, multidomain impairment profiles seen in individuals [8]. Hence, an individualized digital twin brain is expected to model dysfunctions across multiple relevant domains, including their interactions, within a single framework, thereby contributing to a deeper understanding of the pathophysiology and optimal treatment selection.

The third challenge is the gap toward realizing personalized treatment simulations. Significant individual differences in the effectiveness of psychiatric therapies, such as pharmacotherapy and brain stimulation, have been observed even within the same diagnostic group [18]. Predicting the treatment effect for individual patients would lead to an optimized treatment selection. Despite this known individual variability, many existing treatment simulation studies using brain models use approaches based on the average characteristics of a disorder rather than individual characteristics, thus failing to sufficiently account for these differences. Although nascent research aimed at predicting individual treatment effects has begun, such approaches often remain limited [19]. They might predict the impact of specific interventions on brain activity but typically lack a dynamic simulation of how neural changes directly generate subsequent alterations in an individual’s higher-order cognition and resulting behavior [20–22].

In this study, we propose a functional digital twin brain system designed to overcome these challenges. Unlike biophysical models constrained by anatomical structure, our approach leverages generative modeling to learn the mapping from functional connectomes to high-dimensional behavioral dynamics. Our system uses an architecture consisting of 2 components: a “hypernetwork” and a “main network” [23,24]. The hypernetwork uses an individual’s neurobiological connectome data as input and dynamically generates the parameters for the main network. On the basis of these generated parameters, the main network simulates higher-order cognitive processes from sensory input to action generation. This directly integrates individual biological data with cognitive and behavioral dynamics, thereby bridging the first challenge: the gap between biological data and psychiatric symptoms. Furthermore, by incorporating conditioning techniques into the main network, we essentially provided the current task context alongside sensory input to guide processing, enabling a single model architecture per individual to reproduce behavioral and brain activity patterns across multiple tasks representing different functional domains. This facilitates the construction of a digital twin brain model that reflects individual characteristics across multiple domains, thereby addressing the second challenge. Consequently, this integrated model paves the way for personalized treatment simulations, thus tackling the third challenge. Because the connectome is directly linked to real-time brain activity and behavior generation, gradient calculations can be used to explore effective intervention targets (e.g., identifying specific parts of the connectome for intervention) aimed at shifting specific behaviors or brain activities in the desired direction. Moreover, by manipulating the main network, which reflects an individual’s connectome, we can simulate the intervention effects on behavior and brain activity, thereby realizing predictions of personalized behavioral perturbations. In this study, we empirically demonstrate that our proposed system, through the aforementioned approaches, overcomes existing challenges and can potentially make a significant contribution to deepening our understanding of psychiatric disorder mechanisms and advancing the development of personalized therapies.

## Results

### System overview

**Fig. 1.**
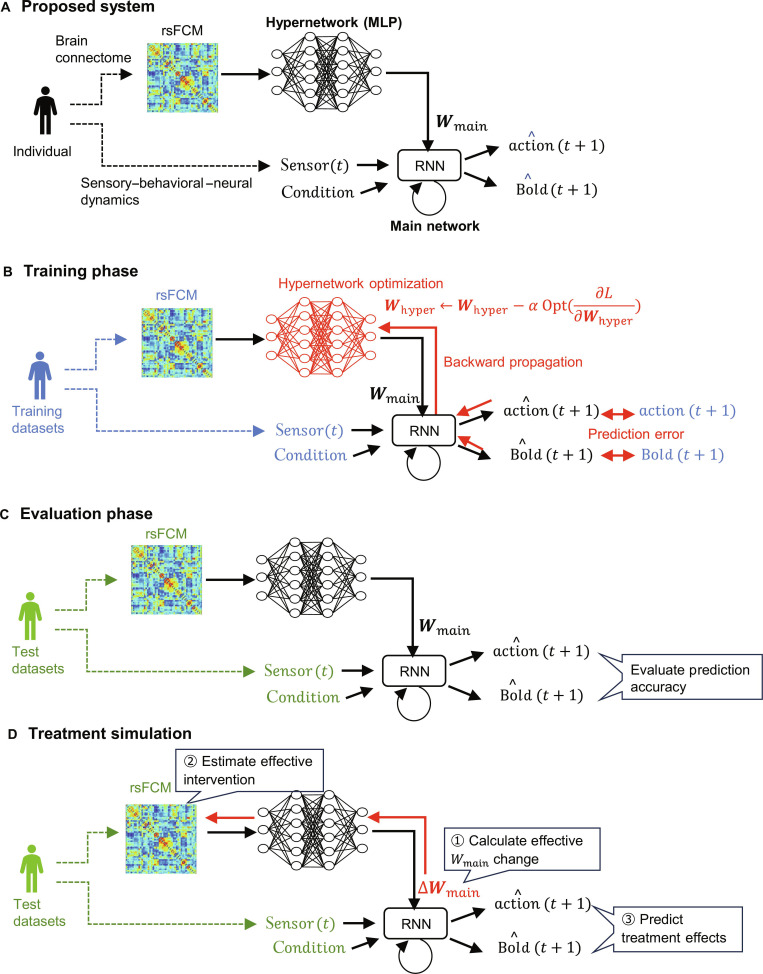
Proposed system and experimental procedure. (A) shows the proposed digital twin brain system, whereas (B) to (D) illustrate the experimental procedure [(B) training phase, (C) evaluation phase, and (D) treatment simulation]. $W_{\text{main}}$ and $W_{\text{hyper}}$ represent the learnable parameters of the main network and hypernetwork, respectively. L is the loss function, α is the learning rate, and Opt is the optimization operator applied to the gradient.

The proposed digital twin brain system (Fig. 1A) comprises 2 main components: a hypernetwork implemented as a multilayer perceptron (MLP) and a main network implemented as a recurrent neural network (RNN). The hypernetwork receives resting-state functional connectivity matrix (rsFCM) data, an indicator of an individual’s brain connectome, and transforms them into a personalized parameter set ($W_{\text{main}}$). These parameters configure every weight and bias in the subsequent main network. The main network receives a joint input comprising the current visual sensory input [$\text{sensor}\left(t\right)$] and a task condition indicator, and it predicts the next-step action $\left[\hat{\text{action}}\left(t+1\right)\right]$ and blood-oxygen-level-dependent (BOLD) signal [$\hat{\text{BOLD}}\left(t+1\right)$], representing neural activity. In this manner, the 2 networks integratively model an individual’s functional connectivity, together with the coupled sensory, behavioral, and neural dynamics that unfold over time.

The rsFCM, along with sensory, action, and BOLD signal data obtained during the tasks used to build the proposed system, was obtained from 228 participants (139 with psychiatric disorders and 89 controls) from the Transdiagnostic Connectome Project (TCP) dataset. Considering the coverage of multiple functional domains defined by the RDoC, we used the Emotional Faces task to assess the Negative Valence Systems domain and the Stroop task to assess the Cognitive Systems domain. The sensory, action, and BOLD signal data from these tasks were prepared to have a common time resolution of 80 ms, which involved interpolating the BOLD signal. The BOLD signal data comprised signals from 20 brain regions distributed throughout the brain. The multimodal task sequences (sensor, action, and BOLD signals) were divided into 2 parts: the first half served as the training dataset and the second half as the test dataset.

For the experimental procedure, in the training phase (Fig. 1B), the hypernetwork was optimized using the training dataset. At each training iteration, the main network, initialized with hypernetwork-generated parameters ($W_{\text{main}}$)​, produced predictions for the forthcoming action and regional BOLD signals. A composite loss L, which is the weighted sum of the prediction errors for the action and BOLD signal targets, was then backpropagated through time. Gradients reached the hypernetwork, compelling it to map the individual’s rsFCM to a set of the main-network parameters ($W_{\text{main}}$) that minimized L. The convergence of the training was confirmed by the monotonic decline in the training loss (Fig. S1). In the subsequent evaluation phase (Fig. 1C), a test dataset is used to verify the generalization performance of the proposed system. This involved evaluating whether the system, using rsFCM and sensor input from the test dataset, could generate action and BOLD signals exhibiting the characteristics of an individual with high accuracy.

Finally, in the treatment simulation (Fig. 1D), we set 2 goals: (a) to identify manipulations of rsFCM that yield intervention effects for each of the multiple functional domains and (b) to predict the behavioral and neural outcomes of applying those manipulations on an individual basis. Intervention efficacy was quantified by changes in the main network’s predicted action sequence and regional BOLD dynamics relative to the preintervention baseline. The detailed framework of the simulation is described later. Briefly, focusing on the fact that the individual dynamics of these actions and BOLD signals are represented in the main network’s weight parameters ($W_{\text{main}}$), we first calculated the optimal change in weight parameters ($\Delta W_{\text{main}}$) required to achieve the best intervention effect. Subsequently, through gradient calculation within the hypernetwork, we identified the specific manipulations on rsFCM designed to achieve the target change in weight parameters ($\Delta W_{\text{main}}$). Finally, we performed virtual intervention by modifying the weight parameters of the main network, subsequently generating new actions and BOLD signals with the altered network, and investigated the individual-specific intervention effects.

### Accuracy of action prediction

**Fig. 2.**
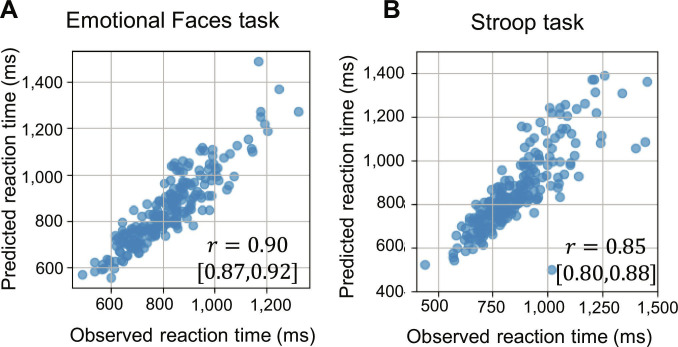
Reproduction of individual differences in reaction times. (A) Reproduction of reaction times in the Emotional Faces task. This scatter plot shows observed reaction times from test data for each individual on the *x* axis (specifically, the time from stimulus presentation to button press). The *y* axis shows reaction times calculated using the same methodology from action sequences predicted by the main network. The high correlation coefficient (*r* = 0.90) indicates that the main network successfully predicts action sequences while reflecting individual differences in reaction times. The 95% confidence interval for the correlation coefficient is provided in parentheses. (B) Reproduction of reaction times in the Stroop task. Similarly, this scatter plot shows observed reaction times on the *x* axis and reaction times calculated from sequences predicted by the main network on the *y* axis. The high correlation coefficient (*r* = 0.85) demonstrates that the main network can reproduce actions that reflect individual differences in reaction times in the Stroop task as well. Combined with the previously mentioned results from the Emotional Faces task, these findings indicate that the unified main network successfully reproduces individual behavioral characteristics across multiple tasks.

We now describe the evaluation of the generalization performance of the action predictions conducted in the evaluation phase. The concordance rate between the action sequences predicted by the model and the participants’ actual choices was high, with a means ± SD of 0.94 ± 0.06 for the Emotional Faces task (2-choice) and 0.90 ± 0.10 for the Stroop task (3-choice). Furthermore, when the reaction times from sensory input to action output were calculated using the predicted sequences, a strong correlation with the observed reaction times was found (*r* = 0.90 for the Emotional Faces task and *r* = 0.85 for the Stroop task; Fig. 2). This demonstrates that the connectome-informed main network accurately predicts actions based on sensory input and captures individual behavioral differences, indicating its ability to reproduce individual-specific, higher-order cognitive functions.

### Accuracy of BOLD signal prediction

**Fig. 3.**
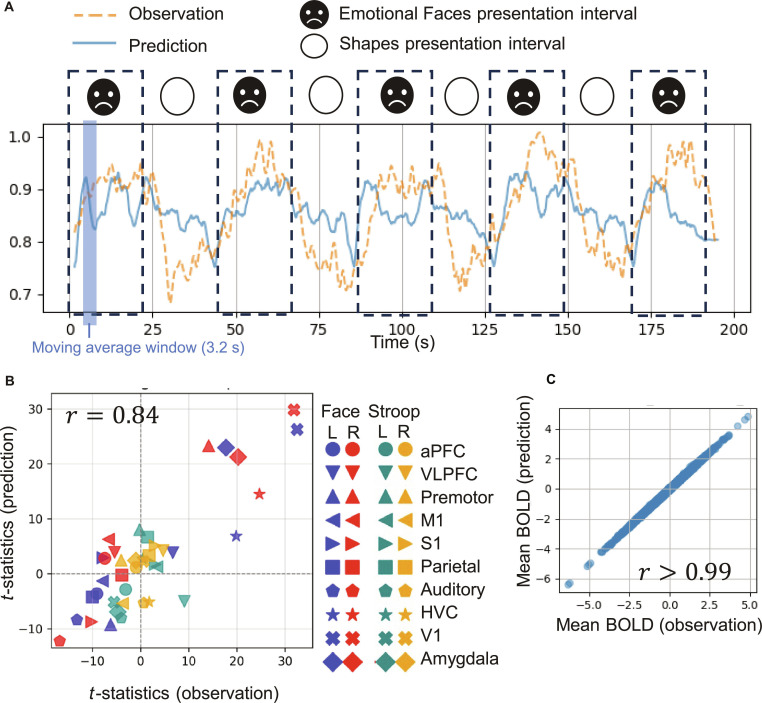
Comparison of observed and predicted BOLD signals. (A) Example of observed and predicted BOLD signals. The plot shows observed and predicted BOLD signals in the left primary visual cortex during the Emotional Faces task, representing a typical example of BOLD signal responses to task stimuli. The predicted BOLD signals are displayed as moving averages over a time window of 3.2 s (40 time steps) to reduce fluctuations. (B) Scatter plot showing results from group-level GLM analysis conducted for each task and brain region to quantitatively evaluate the magnitude of BOLD signal responses to task stimuli. See Materials and Methods for GLM analysis details. The *x* axis shows *t*-statistics (representing neural response strength) calculated from GLM models based on observed BOLD signals, while the *y* axis shows *t*-statistics from GLM models based on BOLD signals predicted by the main network. aPFC, anterior prefrontal cortex; VLPFC, ventrolateral prefrontal cortex; HVC, higher visual cortex. (C) Scatter plot displaying the mean values of observed BOLD signals (*x* axis) versus BOLD signals predicted by the main network (*y* axis), calculated for each individual, brain region, and task.

Having established the model’s ability to predict behavioral actions, we next examined its capacity to capture the underlying neural dynamics by predicting unseen BOLD signal characteristics concurrently with actions. A visual comparison of the predicted and observed BOLD signals (Fig. 3A) revealed similarities and differences. Although time-point level matching is imperfect because of the inherently noisy nature of BOLD data, the predicted signals successfully captured response patterns to the presented task stimuli, such as signals that increased during face presentation and decreased during shape presentation. To move beyond visual assessment and quantitatively evaluate performance in predicting these stimulus-specific response patterns, we computed *t*-statistics (indicating the strength of task-related BOLD responses) using a general linear model (GLM), a standard approach in functional magnetic resonance imaging (fMRI) analysis. This analysis revealed a strong correlation (*r* = 0.84) between the observed and predicted BOLD signals in the task-specific region-wise *t*-statistics (Fig. 3B). These results indicate that the main network accurately captured the BOLD signal characteristics specific to different tasks and brain regions. Crucially, we confirmed that this high prediction accuracy was robust to the preprocessing strategy; results remained consistent when using linear interpolation or when evaluating predictions solely at the original acquisition time points (Fig. S6). Notably, both the observed and predicted data showed high *t*-statistic values in the primary visual cortex, higher visual cortex, and amygdala during the Emotional Faces task, consistent with prior fMRI studies [25–28], and confirming the validity of our model’s predictions. This high correspondence in *t*-statistics, despite imperfect moment-to-moment matching, arises because observed fMRI signals contain both task-evoked neural components and substantial stochastic noise (e.g., physiological and scanner noise). Since our main network is a deterministic model designed to learn systematic input–output relationships, it predicts the “denoised” task-evoked component but does not reproduce the stochastic noise. Therefore, the high correlation in GLM-derived statistics demonstrates that the model successfully captures the underlying neural signal once noise is analytically removed. Furthermore, as shown in Fig. 3C, we found strong correlations between the observed and predicted mean values of the BOLD signals across individuals, demonstrating high accuracy in capturing interindividual differences. Overall, despite not perfectly matching moment-to-moment fluctuations in the BOLD signal, our model effectively captured both individual-specific neural response patterns and task-related activation across brain regions in an unseen test dataset.

### Defining functional indicators and validating intervention directions

Having established the model’s fidelity in capturing individual sensory–behavioral–neural dynamics, we next sought to leverage this generative capability to simulate personalized interventions. Specifically, we aimed to identify connectome manipulations that could modulate targeted cognitive and affective functions, thereby validating the feasibility of our digital twin framework.

In the intervention simulation, we defined 2 functional indicators aimed at functional changes based on the RDoC functional domains. The first indicator, the affective response, was defined as the strength of the amygdala response, represented by the *t*-statistic calculated from a GLM model of BOLD signals during the Emotional Faces task. The second indicator, representing the Cognitive Systems domain, was processing speed, which is defined as the reaction time calculated from action sequences during Emotional Faces and Stroop tasks.

Next, we investigated manipulations to efficiently affect functional indicators (affective response and processing speed). Individual differences in these indicators reflect variations in the main network outputs, which, in turn, are governed by its weight parameters ($W_{\text{main}}$), efficiently affecting the functional indicators required to identify the optimal way to manipulate $W_{\text{main}}$. Accordingly, we identified a unit vector v representing the “direction of change” in $W_{\text{main}}$ that most effectively affects these functional indicators. This vector v, which is interpretable as a coefficient vector indicating the relative contribution of each weight parameter within $W_{\text{main}}$, specifies the pattern of the combined change across these parameters, yielding the largest impact on each functional indicator. To compute this vector v, we used partial least-squares (PLS) regression analysis to analyze the relationship between each functional indicator (dependent variable) and $W_{\text{main}}$ (independent variable) to determine the direction of change most strongly correlated with the changes in functional indicators. The vector that most effectively changed the affective response was defined as $v_{\text{affective}}$ (affective vector), and the vector that most effectively changed the processing speed was defined as $v_{\text{cognitive}}$ (cognitive vector). Thus, these vectors identify the optimal intervention directions.

After mathematically identifying the optimal manipulation vectors, we examined whether these computationally derived vectors were clinically significant. A key question was whether the extent to which an individual’s weight parameters ($W_{\text{main}}$) matched the directionality of these computationally derived vectors ($v_{\text{affective}}$ and $v_{\text{cognitive}}$) is related to their actual clinical presentation. To investigate this, we examined correlations with 107 clinical measures (Table S1) and applied the Benjamini–Hochberg false discovery rate (FDR) correction to account for multiple comparisons. Notably, the cognitive latent variable (derived using corresponding vectors) showed robust statistical significance with domain-specific clinical symptom scores even after correction (Table 1). Specifically, it was positively correlated with cognitive processing speed (digital symbol matching [DSM]: *r* = 0.42, uncorrected $P=3.3\times 10^{-11}$ , $P_{\text{FDR}}=3.3\times 10^{-9}$) and negatively associated with general functional impairment (longitudinal interval follow-up evaluation—range of impaired functioning tool [LIFE-RIFT]: *r* = −0.27, uncorrected $P=1.8\times 10^{-4}$ , $P_{\text{FDR}}=0.010$). Associations with psychiatric symptoms, including positive symptoms and suicidal ideation, showed trends toward significance ($P_{\mathrm{FDR}}$ < 0.15). In contrast, the affective latent variable (derived using corresponding vectors) showed nominally significant associations with emotion-related clinical indices, such as panic attack frequency (panic disorder severity scale [PDSS]: *r* = −0.24, uncorrected $P=1.1\times 10^{-3}$) and reward dependence (temperament and character inventory [TCI]: *r* = −0.20, uncorrected $P=4.5\times 10^{-3}$). While these associations did not survive FDR correction ($P_{\mathrm{FDR}}$ > 0.05), the pattern of correlation—particularly with traits associated with amygdala function—suggests biologically plausible links. Collectively, these results suggest that our approach captures the clinically relevant aspects of psychiatric disorders by encoding meaningful relationships between brain connectomes and functional indicators. This implies that interventions that modify weight parameters in the direction of the identified vectors may have therapeutic potential by acting on the brain connectome underpinning clinical symptoms.

**Table 1.** Top clinical symptoms by absolute correlation with each latent variable

Latent variable	Clinical symptoms	Test name	Correlation coefficient	*P* value	Adjusted *P* value (FDR)
Affective latent variable	Panic attack frequency	PDSS	−0.24	1.1 × 10^−3^	0.118
	Reward dependence	TCI	−0.20	4.5 × 10^−3^	0.241
	Consummatory pleasure	TEPS	−0.18	1.2 × 10^−2^	0.348
	Financial risk perception	DOSPERT	−0.18	1.3 × 10^−2^	0.348
Cognitive latent variable	Processing speed	DSM	0.42	3.3 × 10^−11^	3.3 × 10^−9^
	Functional impairment	LIFE-RIFT	−0.27	1.8 × 10^−4^	0.010
	Positive symptoms	PANSS	−0.21	2.6 × 10^−3^	0.075
	Functional impairment	MCAS	0.21	2.8 × 10^−3^	0.075
	Suicidal behavior	C-SSRS	−0.20	6.9 × 10^−3^	0.148

This table presents the clinical symptoms from the TCP dataset that exhibited the strongest correlations with affective and cognitive latent variables. TEPS, temporal experience of pleasure scale; DOSPERT, domain-specific risk-taking scale; PANSS, positive and negative syndrome scale; MCAS, multidimensional comorbidity assessment scale; C-SSRS, Columbia-suicide severity rating scale.

### Derivation of connectome-based intervention targets

**Fig. 4.**
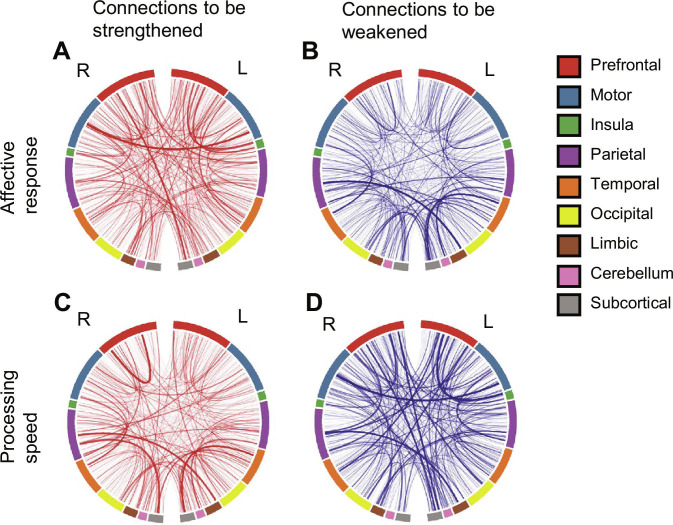
Optimal brain connectivity manipulations for intervention. (A and B) Brain connections that should be strengthened (A) or weakened (B) to affect affective responses. (C and D) Brain connections that should be strengthened (C) or weakened (D) to affect processing speed. To determine the optimal manipulations in resting-state functional connectivity associated with affective responses or processing speed, we first calculated the optimal direction of weight parameter changes in the main network (affective or cognitive vectors). We then derived the necessary resting-state functional connectivity manipulations using gradient calculations (SmoothGrad) within the hypernetwork. See Materials and Methods for further details. This figure shows the average gradient values for all participants. For clarity, only connections with gradient values exceeding 2.5 SDs from the mean are displayed. The line thickness represents the magnitude of the deviation from the mean (SD).

On the basis of the identified optimal manipulation vectors ($v_{\text{affective}}$ and $v_{\text{cognitive}}$), we derived specific rsFCM connections to manipulate and influence the functional indicators (affective response and processing speed). This process of deriving rsFCM manipulation targets can be seen as computationally exploring optimal targets for clinical approaches, such as decoded neurofeedback or neurofeedback, which aim to alter specific brain activity patterns [29,30]. To identify the intervention targets, we utilized gradient backpropagation through the hypernetwork. This allowed us to calculate the adjustments in rsFCM required to induce changes in the weight parameters of the main network ($W_{\text{main}}$) along the affective or cognitive vectors. The results revealed that rsFCM manipulation patterns differed depending on the target functional indicators of the intervention. Strengthening connectivity in bilateral motor cortices (Fig. 4A) and weakening connectivity between limbic and parietal regions and between temporal and subcortical regions (Fig. 4B) affected the affective response. To impact processing speed, we suggested the strengthening connectivity within the prefrontal cortex, between temporal and subcortical regions, and between parietal and subcortical regions (Fig. 4C) and the weakening connectivity between bilateral motor areas (Fig. 4D). The detailed lists of the top 10 connections with the highest gradient magnitudes for each direction (strengthening/weakening) are provided in Tables S2 and S3. These findings demonstrate the potential of this digital twin brain approach for designing precise, domain-specific connectome intervention strategies based on the integrative modeling of an individual’s connectome, neural activity, and action.

### Simulation of individual functional modulations

**Fig. 5.**
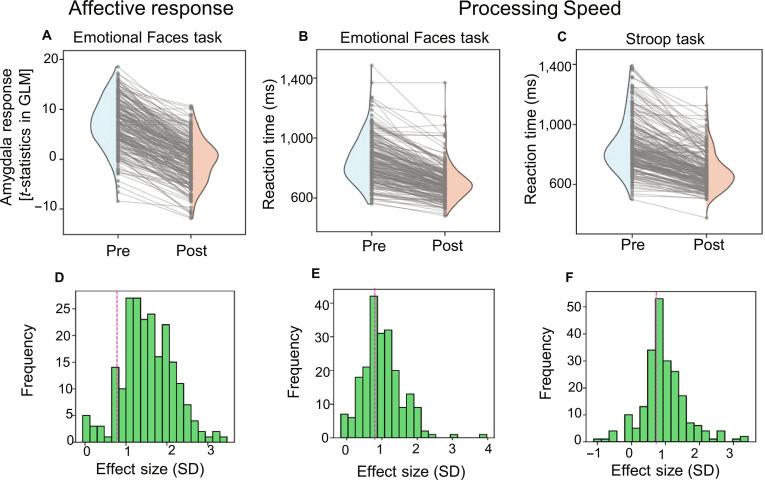
Interventional effects on individual functional indicators. (A) Pre- versus postmanipulation amygdala responses (*t*-statistics; affective response indicator) during the Emotional Faces task. Outward half-violin plots show the pre (left half) and post (right half) distributions; the thin gray lines connect paired pre–post values for each participant, visualizing individual changes in the indicator. (B) Pre- versus postmanipulation reaction times (in milliseconds; processing speed indicator) during the Emotional Faces task. (C) Pre- versus postmanipulation reaction times (in milliseconds) during the Stroop task. (D to F) Histograms showing the distribution of standardized effect sizes across individuals for amygdala responses (D), Emotional Faces task reaction times (E), and Stroop task reaction times (F). Effect sizes were calculated as ([Premanipulation Value] − [Postmanipulation Value]) / Premanipulation SD, such that positive values indicate reduced amygdala response or shortened reaction time. Therefore, effect sizes are in SD units. The dashed vertical lines indicate a large effect size (Cohen’s *d* = 0.8), serving as a benchmark for substantial intervention effects.

To predict responses to personalized perturbations, we simulated the effects of the identified manipulations on individual participants. For each participant, the weight parameters of the main network were modified along the direction of the affective or cognitive vectors. Using these parameters, a postmanipulation main network was constructed to generate new predictions of the action and BOLD signals in response to the sensor input. A comparison of the functional indicators (affective response and processing speed) calculated from these predicted actions and BOLD signals before and after the manipulation (Fig. 5A to F) revealed intervention effects in most individuals. Manipulation targeting the affective response reduced the amygdala response strength (Fig. 5A), while manipulation targeting processing speed shortened reaction times (Fig. 5B and C). Importantly, substantial interindividual variability was observed in the magnitude of these intervention effects (Fig. 5D to F); individual effect sizes ranged widely, from negligible (approximately 0 SD) in some participants to large (>2 SD) in others. This heterogeneity in simulated outcomes, primarily arising from individual differences in baseline brain connectomes, mirrors clinical response variability and suggests that our digital twin brain approach could help predict individual treatment efficacy before intervention.

## Discussion

In this study, we demonstrated that a novel digital twin brain framework can successfully predict individual behavioral responses and simulate personalized treatment effects in psychiatric disorders. Our framework integrates individual biological brain connectomes (rsFCM) with sensory–behavioral–neural dynamics during task performance, thereby creating a high-fidelity model of personalized higher-order brain functions. This model synergistically combines a hypernetwork that captures static brain connectomes with a main network that sequentially generates actions and BOLD signals from sensory inputs. This integration enables our framework to transcend the limitations of previous personalized brain models, which primarily focus on replicating resting-state or simple sensory processing, by simulating dynamic brain activity and behavior during multitask performance, spanning both affective response and cognitive processing domains within a unified architecture. This integrative approach provides a unified framework for individual differences in functional domains relevant to psychiatric conditions while establishing a direct link between individual biological characteristics and behavior for treatment simulations.

The main network of our digital twin brain system successfully predicted behavior in response to task stimuli with high accuracy. The prediction accuracy for individual response choices across Emotional Faces and Stroop tasks was high, with concordance rates of 0.90 to 0.94. Corresponding correlation coefficients, calculated for comparison with previous studies, were 0.88 to 0.89, notably exceeding the range (0.16 to 0.57) reported in those studies, which predicted behavior and cognitive scores from resting-state connectivity [31–35]. Furthermore, the correlation between observed and predicted reaction times across these tasks demonstrated high value ranges (0.85 to 0.90), outperforming previous research on reaction time prediction from neurophysiological indicators, which reported correlation coefficients of 0.09 to 0.80 [36,37]. These high prediction accuracies demonstrate the effectiveness of our approach, in which the hypernetwork generates personalized parameters for the main network based on individual rsFCM data, thereby enabling the main network to capture and reproduce participant-specific behavioral characteristics.

We further analyzed the relationship between model predictions and participant baseline performance. First, we found that the model–participant concordance rate was positively correlated with baseline accuracy (*r* > 0.76), implying that model performance depends on the individual’s competency level (Fig. S2). Second, the distribution of concordance rates suggested that the model successfully learned the individual’s systematic tendency to commit errors (i.e., error probability) rather than overfitting to the specific timing of stochastic errors. Third, the model’s average accuracy closely matched that of the participants (e.g., emotion: 0.96 versus 0.97), confirming that the proposed model does not act as an idealized agent that solves tasks perfectly but rather faithfully replicates the specific level of cognitive performance of each individual (Fig. S3).

To further evaluate the model’s potential for broad applicability, we tested its performance on unseen participants (cross-participant generalization; see Table S4 and Methods S1). The model successfully predicted action choices (>87% accuracy) and BOLD patterns (*r* > 0.83) for new individuals based solely on their connectomes. However, the prediction of reaction times for unseen participants was less precise (*r* ≈0.36 to 0.42) compared to the model generalized within the same individual (*r* > 0.85). While these values are comparable to or exceed typical connectome-to-behavior correlations reported in previous studies [38,39], the drop in performance highlights the difficulty of inferring fine-grained temporal dynamics exclusively from static connectomes with the current data scale. We clarify that, at this stage, this level of prediction accuracy remains a research limitation regarding immediate clinical utility for patient stratification. We suggest that future work utilizing larger datasets may help address this challenge, potentially enabling precise behavioral prediction directly from the connectome without the need for within-individual training.

Despite the relatively large number of parameters in the main network compared to the sample size, our multifaceted regularization strategy effectively mitigated overfitting. Specifically, the hypernetwork’s bottleneck architecture significantly reduces the effective degrees of freedom by generating weights from a lower-dimensional latent space. This implicit regularization, combined with explicit dropout and data augmentation via sequence segmentation, ensured that the model learned robust, generalized dynamics rather than memorizing noise. Consequently, the model achieved high generalization performance on unseen test data within individuals and robustly reproduced population-shared features (e.g., action choices and BOLD patterns) in cross-participant evaluations. However, capturing highly variable individual traits such as reaction times in a zero-shot setting remains a challenge for future scaling.

To validate the necessity of the hypernetwork architecture, we critically compared it against alternative modeling approaches, focusing on the distinction between shared versus individualized weight spaces. First, theoretical considerations suggest that treating connectome features as additive inputs to a shared network (as in standard RNNs) merely provides a static bias shift $\left[h_{t}=\sigma \left(W_{\text{in}}x_{t}+W_{\mathrm{rec}}h_{t-1}+W_{c}c+b\right)\right]$, whereas the hypernetwork allows the connectome to modulate the weights multiplicatively $\left[h_{t}=\sigma \left(W_{\text{in}}\left(c\right)x_{t}+W_{\mathrm{rec}}\left(c\right)h_{t-1}+b\right)\right]$. We hypothesize that psychiatric heterogeneity involves alterations in circuit dynamics that require this multiplicative modulation, consistent with studies highlighting the superior representational power of multiplicative interactions [40,41]. Indeed, our empirical comparison with a “direct input” model confirmed that additive inputs were less effective in capturing individual behavioral variability in our task setting (e.g., reaction time correlation *r* ≈ 0; see Fig. S4, Table S5, and Methods S2). Second, while fine-tuning models with individual behavioral data is a powerful strategy for personalization [42], it requires collecting extensive task performance data from each patient. In contrast, our hypernetwork offers the potential for “zero-shot” generation of personalized models solely from resting-state connectomes (similar to one-shot learning approaches [43]). While further scaling of data may be required to fully realize this potential for all behavioral metrics, this capability is essential for our goal of designing gradient-based interventions without preexisting behavioral data.

As demonstrated by the model’s ability to predict performance across both the Emotional Faces and Stroop tasks, embedding these tasks in a single generative model confers distinct advantages over the conventional “one-task-per-model” paradigm. First, it elucidates context-dependent interactions between functional domains. By modulating the shared connectome-derived parameters, we observed that interventions targeting affective responses did not generalize to processing speed during the affect-neutral Stroop task (*r* < 0.01) but showed a positive coupling during the Emotional Faces task (*r* = 0.12; Fig. S5). This reveals that affective circuits may modulate cognitive processing speed specifically under negative valence contexts—a mechanistic nuance invisible to single-task models. Second, a unified architecture is a prerequisite for multiobjective optimization. Since clinical cases often involve co-occurring deficits, our framework allows for gradient-based searches that optimize global therapeutic outcomes across multiple domains, identifying interventions that balance trade-offs rather than improving one symptom at the expense of another. While our framework enables such multiobjective optimization, our results also highlighted the heterogeneity of patient responses. Simulating individual intervention effects via manipulations confirmed significant interindividual variability in treatment response, a phenomenon also commonly seen in clinical practice [18,44,45]. In the context of our digital twin brain system, several factors may contribute to the observed individual differences: (a) variations in individual rsFCM patterns; (b) ceiling effects due to differences in baseline reaction times and amygdala responses; (c) the direction of affective and cognitive vectors extracted by PLS being optimized at the population level and not necessarily optimal for each individual; and (d) the nonlinearity of the main network weight parameter space. These findings underscore the need to predict individual treatment effects and highlight future challenges in optimizing parameters for each individual.

There has been growing interest in modeling individual neural activity dynamics and their relationship with higher-order brain functions in recent years [10,11,46,47]. However, many of these studies have been limited to analyzing the correlations between modeled brain activity and behavioral metrics, without capturing the sequential generation of brain activity and behavior in response to sensory inputs, or their interactive dynamics. Furthermore, while studies by van Bueren et al. [19] and Sun et al. [48] have attempted to optimize stimulation interventions for individual brain networks, these efforts have primarily focused on inducing specific brain activities, with limited evaluation of higher-order functions involving sensory processing and behavior. In contrast, our study extends these previous approaches by developing a digital twin system that integrates individual static connectomes, captures sensory–behavioral–neural dynamics, and enables the simulation of intervention effects. This integrated approach highlights the novel contribution of our research in understanding complex psychiatric disorders and developing personalized treatment strategies.

Furthermore, we identified connectome changes through gradient calculations within the hypernetwork that efficiently affected specific functional indicators in the model. For the affective domain, manipulations weakening connections between the limbic, subcortical, parietal, and temporal regions were associated with impacts on affective responses, supporting previous research suggesting that networks centered around the amygdala in the limbic system may trigger excessive emotional responses [49–52]. For the cognitive domain, connections between the prefrontal cortex, motor cortex, parietal lobe, and subcortical regions have been suggested to contribute to processing speed, which aligns with existing fMRI studies indicating that these networks are important attentional networks for processing speed [8,53–56]. These findings suggest the possibility of individualizing manipulation strategies that target specific functional indicators across cognitive and affective domains.

It is important to distinguish between the clinical validity of our target parameters and the generative nature of our simulations. The correlations between model-derived latent vectors and clinical scores (Table 1) serve to validate that the model captures biologically relevant dimensions of psychopathology. Building on this validation, our in silico perturbation experiments (Fig. 5) demonstrate a direct generative link: specific manipulations of the connectome input result in directional changes in behavioral and neural outputs. While these “functional modulations” do not directly equate to clinical cures, they provide a mechanistic framework for identifying candidate interventions that can perturb the brain system from a pathological state toward a desired functional profile.

However, we acknowledge that translating these in silico connectome manipulations into physical interventions poses challenges. Current noninvasive brain stimulation techniques (e.g., transcranial magnetic stimulation) or neurofeedback cannot yet manipulate individual functional connections with the granular precision simulated in our model. Nevertheless, identifying these optimal connectivity patterns serves as a crucial theoretical roadmap for future therapy. Furthermore, emerging evidence from lesion network mapping and stimulation studies suggests that focal interventions on specific “hub” regions can propagate to induce widespread network-level reorganization [22,57,58]. Thus, the complex connectivity changes predicted by our digital twin might be practically realizable by targeting a few key nodes that drive these desired global network dynamics, rather than manipulating each connection individually.

Our study has some limitations. First, the sample size (139 individuals with psychiatric disorders and 89 healthy controls) might not have fully captured the diversity and individual differences under psychiatric conditions. Second, the tasks were limited to Emotional Faces and Stroop paradigms. In addition, because of dataset constraints, we evaluated generalization using a temporal split within a single session; future research utilizing longitudinal datasets is required to verify the model’s generalization across different days and sessions. Third, our analyses relied on functionally defined brain atlases (Schaefer, Tian, and Buckner atlases) as provided by the preprocessed dataset. While these atlases ensure high functional homogeneity within regions, future studies should assess the model using structurally defined atlases (e.g., Desikan–Killiany or Brainnetome) to evaluate the robustness of the findings across different parcellation schemes, as parcellation choice is known to influence connectome-based prediction performance [59], and to better elucidate the relationship between anatomical constraints and functional dynamics. Fourth, this study primarily focused on the mesoscopic scale, examining connectivity between brain regions, whereas the brain exhibits a multiscale structure from the molecular and cellular levels to large-scale networks [9,60–62]. Incorporating microscopic models (at the ion channel and receptor levels) could potentially extend our approach to simulating pharmacological interventions and other treatment modalities.

Despite these limitations, the digital twin brain system developed in this study has significant potential for future advancement. A key strength of our model, compared to conventional individual brain models, is its ability to learn flexibly using artificial neural networks. As it uses sensory inputs and behavioral outputs similar to the actual brain, future work could potentially reproduce sensory and behavioral input–output patterns not only in specific cognitive and emotional tasks but also throughout an individual’s daily life activities. Although we have adopted simple RNNs to capture the sequential nature of brain processing, there is value in exploring more advanced models, such as long short-term memory (LSTM) networks, gated recurrent units (GRUs), graph neural networks and transformers (and emerging state space models), for modeling large-scale data and long-term dependencies. Furthermore, by developing methods to analyze the structure of individual parameter spaces and search for optimal intervention directions tailored to each person, we expect to pave the way for personalized medicine adapted to psychiatric symptoms and diverse cognitive characteristics. In addition, integrating microscopic level modeling could enable more comprehensive simulations that incorporate the effects of pharmacological therapies and genetic factors. Through these extensions, the digital twin brain system presented in this study is expected to serve as an increasingly powerful platform for elucidating the pathophysiology of psychiatric disorders and predicting individualized treatments.

## Materials and Methods

### Dataset

We used data from the publicly available TCP dataset [63]. The TCP dataset consists of 241 participants, including individuals with a wide range of mental disorders and healthy controls, recruited between 2019 November and 2023 March. We accessed preprocessed BOLD signal data, parcellated by brain region, and the corresponding behavioral data. For this study, we used data from 228 participants (139 with mental disorders and 89 healthy controls) for whom resting-state BOLD signals, task-related BOLD signals, behavioral data, and clinical information were available. The age of the participants ranged from 18 to 68 years (mean = 34.0, SD = 13.0), with 97 males and 128 females included. Data for the Emotional Faces task were available for 95.6% (*n* = 218) of the participants, and data for the Stroop task were available for 98.2% (*n* = 224) of the participants. Participants whose accuracy rates were below the chance level on the Emotional Faces task (*n* = 5) and Stroop task (*n* = 5) were excluded from the reaction time and accuracy analyses for the respective tasks.

For all participants, BOLD signals, sensory inputs, and action data were recorded for 394.6 s during the Emotional Faces task and 407.2 s during the Stroop task. These data were split into training and testing sets for the training and evaluation of the digital twin system. For each task, the first 196.8 s of data were used as the training set, and the subsequent 196.8 s of equal length was used as the testing set. This temporal division allowed us to evaluate the model’s ability to predict behavior and brain activity in response to test sensory inputs, thereby enabling us to assess within-participant generalization capability.

This secondary analysis of deidentified data was approved by the Institutional Review Board of the National Center of Neurology and Psychiatry (IRB no. A2021-119).

### Tasks

We utilized 2 standard fMRI paradigms to probe distinct functional domains.

• Emotional Faces task: To assess the Negative Valence Systems domain, we used a matching task adapted from Hariri et al. [25]. Participants matched a target image (either an emotional face or a geometric shape) to 1 of 2 probe images. We focused on trials involving negative facial expressions (e.g., anger and fear) to elicit amygdala reactivity [26,64].
• Stroop task: To assess the Cognitive Systems domain (specifically cognitive control and processing speed), we used a classic color-word Stroop task [65]. Participants identified the ink color of color-words presented under either congruent (matched) or incongruent (mismatched) conditions. The Stroop effect, characterized by reaction time interference in incongruent trials, serves as a robust measure of cognitive flexibility and inhibition [66,67].

### fMRI data preprocessing in public datasets

For the public dataset (TCP), fMRI data acquisition, preprocessing (including noise removal), and brain region parcellation were performed following the standard procedures. For detailed information, please refer to the data release paper [63]. MRI data were acquired at both facilities using calibrated Siemens 3 T MAGNETOM Prisma scanners with 64-channel head coils. All fMRI runs adhered to the Human Connectome Project (HCP) protocol, consisting of 488, 510, and 493 volumes for the resting state, Stroop task, and Emotional Faces task, respectively. The common fMRI sequence parameters were repetition time (TR) = 800 ms, echo time = 37 ms, flip angle = 52°, voxel size = 2 mm, and multiband acceleration factor = 8. Functional scan slices were automatically aligned parallel to the anterior–posterior (AP) commissure plane and centered on the brain. B0 field maps in the AP/posterior–anterior directions were acquired for distortion correction; however, this study utilized AP direction data from the resting state, Emotional Faces task, and Stroop task.

MRI data were preprocessed using the HCP Pipeline [68] (version 4.7.0), involving minimal processing and noise removal. Tools from the FMRIB Software Library and FreeSurfer [69] were primarily utilized. Structural MRI processing included the alignment of T1-weighted/T2-weighted images from each participant’s native space to the Montreal Neurological Institute (MNI) space, correction for distortion and inhomogeneity, surface registration based on FreeSurfer and Conte69 atlases, and downsampling to 2 mm. The fMRI volume processing involves spatial distortion correction, motion correction (FMRIB’s Linear Image Registration Tool registration), bias field correction, and normalization. For fMRI surface processing, 4-dimensional volume time-series data were converted to standard gray ordinate surface data and aligned along the tissue contours using HCP algorithms. Surface data were smoothed and aligned across participants using MSMAll [70], which utilizes features such as cortical folding. In addition, ICA-FIX [71] was applied to remove noise sources, such as motion and scanner drift, using classifiers pretrained on HCP data. Global signal regression has also been implemented for noise control in fMRI time-series data and improvement of action prediction models.

To calculate the functional connectivity, dense CIFTI time-series data with 91,282 vertices were parcellated into 446 brain regions encompassing the cortical, subcortical, and cerebellar areas. This was accomplished by averaging the functional time-series data within each region. Cortical parcellation utilizes a 400-region surface-based functional atlas [72]. The subcortical regions were parcellated into 32 laterally symmetric regions using the atlas of Tian et al. [73]. The cerebellum was parcellated into 14 regions using the atlas of Buckner et al. [74].

### fMRI data preprocessing for experiments

To create rsFCMs, we utilized correlation matrices between 446 brain regions from the publicly available dataset. Specifically, we calculated the Pearson correlation coefficients between regions from each participant’s BOLD signal data. The upper triangular elements of the resulting correlation matrices were extracted and transformed using Fisher’s *z*-transformation, representing the rsFCMs as vectors with 99,235 elements.

For BOLD signal data during the Emotional Faces and Stroop tasks, we selected 20 brain regions that provided comprehensive coverage of the brain to reduce computational costs. These regions included the bilateral anterior prefrontal cortex, ventrolateral prefrontal cortex, premotor cortex, primary motor cortex (M1), primary sensory cortex (S1), superior parietal cortex, auditory cortex, higher visual cortex, primary visual cortex (V1), and amygdala (Table S6).

Regarding temporal resolution, while the original BOLD signal data had a TR of 0.8 seconds, we resampled the data to 0.08-s (10-fold) intervals using cubic interpolation to align the temporal intervals with sensory and behavioral data. We emphasize that this resampling does not increase the intrinsic temporal resolution of the BOLD signal. To assess robustness against potential interpolation-induced artifacts, we performed 2 additional validation analyses: (a) GLM analyses using only predicted BOLD values sampled at the original acquisition time points (TR = 0.8 s), excluding all interpolated frames, and (b) full model retraining using linear interpolation instead of cubic interpolation. Results of these analyses are reported in Fig. S6 and demonstrate that prediction performance and spatial response patterns are robust to the choice of interpolation scheme.

Finally, for data normalization, we calculated the mean and SD across all participants and time steps for each brain region in the training data and applied *z*-score normalization. The means and SDs derived from the training data were used to normalize the test data.

### Preprocessing of sensory, task condition, and action data

We prepared sensory inputs, task condition inputs, and action outputs from Emotional Faces and Stroop tasks as time-series data suitable for training the main network model. The time interval (temporal resolution) of the time-series sequences was set at 0.08 s to represent individual differences in reaction time and to align with the original TR of 0.8 s in the fMRI data. Sensory inputs are represented in 8 dimensions, task conditions in 1 dimension, and action outputs in 3 dimensions.

Sensory inputs represented the visual stimuli in 8 dimensions for both tasks. In the Emotional Faces task, these 8 dimensions were fully utilized. First, we used 2 dimensions to represent cue stimuli (“match faces” or “match shapes”). During cue presentation, a value of “1” was input to the corresponding dimension and “0” when not presented. Next, we represented the facial stimuli at the top, bottom left, and bottom right of the screen using 2 dimensions each, for a total of 6 dimensions. When no facial stimuli were present, all 6 values were set to “0”; during presentation, values drawn from a uniform distribution of [0, 0.1] or [0.9, 1] were used to represent individual and emotional differences in facial expressions. Regarding shape stimuli (ellipses), when none were present, all 6 values corresponding to the 3 locations were similarly set to “0”. During shape presentation, information about the ellipses’ different orientations was encoded using combinations of “1” and “0”. This information was assigned to the same 6-dimensional elements corresponding to the top, bottom-left, and bottom-right locations used for the facial stimuli (2 dimensions per location). In the Stroop task, 6 of 8 dimensions were used to represent visual stimuli. Specifically, 3 dimensions represented the color of the presented characters (red, green, and blue), and 3 dimensions represented the text of the characters (red, green, and blue). When no characters were presented, these 6 dimensions were set to “0”; during presentation, a value of “1” was input to the dimension corresponding to the presented color or text. The remaining 2 dimensions of the sensory input were always set to “0” during the Stroop task.

The task condition input, which remained constant over time for each respective task, was a one-dimensional identifier set to “0” during the Emotional Faces task and “1” during the Stroop task.

Action outputs were represented in 3 dimensions for both tasks. In the Emotional Faces task, 2 dimensions were used: one for left button presses and another for right button presses. When a button was pressed, a value of “1” was assigned to the corresponding dimension, and “0” otherwise. The third dimension was always set to “0”. In the Stroop task, all 3 dimensions were utilized: one dimension for each of the red, green, and blue button presses. When a button was pressed, a value of “1” was assigned to the corresponding dimension, and “0” otherwise.

### Clinical symptom measures

As part of the treatment intervention simulation, correlations were examined between the affective and cognitive vectors derived from the model and the clinical symptom scores of the actual participants. These clinical symptom scores were included in the TCP dataset and assessed using an evaluation battery that was administered across 3 testing sessions. The evaluation battery encompassed a wide range of domains, including function, lifestyle, emotion, mental health, cognition, environment, personality, and social factors. These include multiple commonly used clinical tools, scales that capture unique aspects of experience, general cognitive function measurements, and self-report scales. The specific clinical measures investigated for correlations with the affective and cognitive vectors are listed in Table S1.

### Model overview

The proposed digital twin brain system comprises a dual-component architecture consisting of a hypernetwork and a main network, designed to reproduce brain dynamics specific to an individual. The hypernetwork takes the brain connectome represented by each participant’s rsFCM as input and uses an MLP to generate the weight and bias parameters for the main network ($W_{\text{main}}$). The main network, based on individual-specific parameters supplied by the hypernetwork, receives a combined input of the sensory input $\text{sensor}\left(t\right)$ and the task condition at each time point and sequentially updates its internal hidden state to simultaneously output the predicted action $\hat{\text{action}}\left(t+1\right)$ and predicted BOLD signal $\hat{\text{BOLD}}\left(t+1\right)$ for the next time point. This model was trained end to end under a loss function that included prediction errors for both actions and BOLD signals. Overall, this system offers a flexible and efficient framework for directly predicting individual brain dynamics based on participant-specific network properties.

### Hypernetwork

The hypernetwork [23,24], which generates parameters for the main network, consists of an MLP. The input layer contains $N_{\text{in}}$ units and receives an input vector $x_{\text{hyper}}^{\left(i\right)}$ ($x_{\text{hyper}}^{\left(i\right)}\in {\mathbb{R}}^{N_{\text{in}}}$), which represents the rsFCM of the participant i. To prevent overfitting, a dropout layer with a rate d is applied to the first layer. This is followed by 2 hidden layers, each containing $N_{\text{hidden}\_\text{hyper}}$ units with Mish activation functions. The final output layer contains $N_{\text{params}\_\text{main}}$ units, which correspond to the total number of learnable parameters in the main network, as described below.

The computational flow through the hypernetwork can be expressed by the following equations:

$$h_{1}^{\left(i\right)}=\text{Mish}\left(W_{1}x_{\text{hyper}}^{\left(i\right)}+b_{1}\right)$$ (1)

$$h_{2}^{\left(i\right)}=\text{Mish}\left(W_{2}h_{1}^{\left(i\right)}+b_{2}\right)$$ (2)

$$y_{\text{hyper}}^{\left(i\right)}=W_{3}h_{2}^{\left(i\right)}+b_{3}$$ (3)

where $h_{1}^{\left(i\right)}$ ($h_{1}^{\left(i\right)}\in {\mathbb{R}}^{N_{\text{hidden}\_\text{hyper}}}$) is the output of the first fully connected layer, $h_{2}^{\left(i\right)}$ ($h_{2}^{\left(i\right)}\in {\mathbb{R}}^{N_{\text{hidden}\_\text{hyper}}}$) is the output of the second fully connected layer, and $y_{\text{hyper}}^{\left(i\right)}$ ($y_{\text{hyper}}^{\left(i\right)}\in {\mathbb{R}}^{N_{\text{params}\_\text{main}}}$) is the final output. The weights and biases for each layer are defined as follows: $W_{1}\in {\mathbb{R}}^{N_{\text{hidden}\_\text{hyper}}\times N_{\text{in}}}$ and $b_{1}\in {\mathbb{R}}^{N_{\text{hidden}\_\text{hyper}}}$ for the first fully connected layer; $W_{2}\in {\mathbb{R}}^{N_{\text{hidden}\_\text{hyper}}\times N_{\text{hidden}\_\text{hyper}}}$ and $b_{2}\in {\mathbb{R}}^{N_{\text{hidden}\_\text{hyper}}}$ for the second fully connected layer; and $W_{3}\in {\mathbb{R}}^{N_{\text{params}\_\text{main}}\times N_{\text{hidden}\_\text{hyper}}}$ and $b_{3}\in {\mathbb{R}}^{N_{\text{params}\_\text{main}}}$ for the output layer.

The Mish activation function is defined as follows:

$$\text{Mish}\left(u\right)=u\cdot \tanh\left(\text{softplus}\left(u\right)\right)$$ (4)

$$\text{softplus}\left(u\right)=\text{ln}\left(1+\text{exp}\left(u\right)\right)$$ (5)

Through these operations, the hypernetwork generates the main network parameters from the individual rsFCMs, enabling personalized modeling.

### Main network

The main network consisted of an RNN and fully connected layers designed for time-series data processing. At each time step t, the combined input of sensory information and task condition, denoted as $x_{\text{main}}^{\left(i\right)}\left(t\right)$, for participant i is fed into a simple RNN with $N_{\text{hidden}\_\text{main}}$ hidden units. We selected a standard vanilla RNN architecture to serve as a parsimonious model of neural population dynamics (firing rates), a widely adopted approach in computational neuroscience [75,76], favoring its simplicity and established biological interpretation over more complex gated architectures like LSTMs or GRUs. We denote the hidden state of this RNN at time t by $h_{\text{main}}^{\left(i\right)}\left(t\right)\in {\mathbb{R}}^{N_{\text{hidden}\_\text{main}}}$, which is updated according to

$$h_{\text{main}}^{\left(i\right)}\left(t\right)=\text{tanh}\left(W_{\mathrm{ih}}^{\left(i\right)}x_{\text{main}}^{\left(i\right)}\left(t\right)+b_{\text{ih}}^{\left(i\right)}+W_{\mathrm{hh}}^{\left(i\right)}h_{\text{main}}^{\left(i\right)}\left(t-1\right)+b_{\text{hh}}^{\left(i\right)}\right).$$ (6)

Here, $W_{\text{ih}}^{\left(i\right)}\in {\mathbb{R}}^{N_{\text{hidden}\_\text{main}}\times N_{\text{sensor}}}$ and $b_{\text{ih}}^{\left(i\right)}\in {\mathbb{R}}^{N_{\text{hidden}\_\text{main}}}$ are the weight matrix and bias vector from input to hidden layer, while $W_{\mathrm{hh}}^{\left(i\right)}\in {\mathbb{R}}^{N_{\text{hidden}\_\text{main}}\times N_{\text{hidden}\_\text{main}}}$ and $b_{\text{hh}}^{\left(i\right)}\in {\mathbb{R}}^{N_{\text{hidden}\_\text{main}}}$ are the weight matrix and bias vector from hidden to hidden layer.

The hidden state $h_{\text{main}}^{\left(i\right)}\left(t\right)$ is then input into a fully connected layer to obtain the final output $y_{\text{main}}^{\left(i\right)}\left(t+1\right)$. From this output vector $y_{\text{main}}^{\left(i\right)}\left(t+1\right)$, we obtain the predicted action ${\hat{\text{action}}}^{\left(i\right)}\left(t+1\right)$ and BOLD signals ${\hat{\text{BOLD}}}^{\left(i\right)}\left(t+1\right)$ by appropriately dividing its dimensions. Formally,

$$y_{\text{main}}^{\left(i\right)}\left(t+1\right)=\left[\begin{array}{c} {\hat{\text{action}}}^{\left(i\right)}\left(t+1\right)\\ {\hat{\text{BOLD}}}^{\left(i\right)}\left(t+1\right) \end{array}\right]\in {\mathbb{R}}^{N_{\text{action}}+N_{\text{BOLD}}},$$ (7)

where $N_{\text{action}}$ and $N_{\text{BOLD}}$ are the dimensions of the action and BOLD signals, respectively. The fully connected layer computed

$$y_{\text{main}}^{\left(i\right)}\left(t+1\right)=W_{\mathrm{ho}}^{\left(i\right)}h_{\text{main}}^{\left(i\right)}\left(t\right)+b_{\mathrm{ho}}^{\left(i\right)}$$ (8)

where $W_{\text{ho}}^{\left(i\right)}\in {\mathbb{R}}^{\left(N_{\text{action}}+N_{\text{BOLD}}\right)\times N_{\text{hidden}\_\text{main}}}$ and $b_{\text{ho}}^{\left(i\right)}\in {\mathbb{R}}^{N_{\text{action}}+N_{\text{BOLD}}}$ are the weight matrix and bias vector from hidden to output layer, respectively.

The output of the hypernetwork $y_{\text{hyper}}^{\left(i\right)}$ corresponds to a concatenated vector of all learnable parameters in the main network ($W_{\mathrm{ih}}^{\left(i\right)}$, $b_{\mathrm{ih}}^{\left(i\right)}$, $W_{\mathrm{hh}}^{\left(i\right)}$, $b_{\mathrm{hh}}^{\left(i\right)}$, $W_{\mathrm{ho}}^{\left(i\right)}$, $b_{\mathrm{ho}}^{\left(i\right)}$), where $N_{\text{params}\_\text{main}}$ represents the total number of these parameters. The matrix formed by vertically stacking these concatenated vectors of all learnable parameters in the main network for all participants is denoted as $W_{\text{main}}$ ($W_{\text{main}}\in {\mathbb{R}}^{N_{\text{participants}}\times N_{\text{params}\_\text{main}}}$), where $N_{\text{participants}}$ is the total number of participants.

### Loss function

For network training, we used a combined loss function that incorporated 2 objectives: action prediction and BOLD signal prediction. We applied the binary cross-entropy loss for the action prediction component and the mean squared error loss for the BOLD signal prediction component, with each loss weighted by a coefficient and then summed.

The loss function for action prediction, denoted as $L_{\text{action}}$, is defined for predicted actions ${\hat{\text{action}}}^{\left(i\right)}\left(t\right)$ and observed actions ${\text{action}}^{\left(i\right)}\left(t\right)$ as follows:

$$\begin{array}{c} \begin{array}{c} L_{\text{action}}=\lambda_{\text{action}}\frac{1}{N_{\text{batch}}\cdot T}\sum_{i=1}^{N_{\text{batch}}}\sum_{t=1}^{T}\\ \left[-{\text{action}}^{\left(i\right)}\left(t\right)\text{log}\left({\hat{\text{action}}}^{\left(i\right)}\left(t\right)\right)-\left(1-{\text{action}}^{\left(i\right)}\left(t\right)\right)\text{log}\left(1-{\hat{\text{action}}}^{\left(i\right)}\left(t\right)\right)\right] \end{array} \end{array}$$ (9)

Here, $\lambda_{\text{action}}$ represents the weighting coefficient for the action prediction loss. The summation is calculated across all participants i and time steps t, where $N_{\text{batch}}$ is the number of participants per batch and T is the number of time steps.

The loss function for BOLD signal prediction, denoted as $L_{\text{BOLD}}$, is defined for the predicted BOLD values ${\hat{\text{BOLD}}}^{\left(i\right)}\left(t\right)$ and observed BOLD values ${\text{BOLD}}^{\left(i\right)}\left(t\right)$ as follows:

$$L_{\text{BOLD}}=\lambda_{\text{BOLD}}\frac{1}{N_{\text{batch}}\cdot T}\sum_{i=1}^{N_{\text{batch}}}\sum_{t=1}^{T}{\left({\hat{\text{BOLD}}}^{\left(i\right)}\left(t\right)-{\text{BOLD}}^{\left(i\right)}\left(t\right)\right)}^{2}$$ (10)

Here, $\lambda_{\text{BOLD}}$ represents the weighting coefficient for the BOLD signal prediction loss. The total loss function L is defined as the sum of the action prediction loss and the BOLD signal prediction loss.

$$L=L_{\text{action}}+L_{\text{BOLD}}$$ (11)

This equation was used to optimize the model by minimizing the prediction errors for both action prediction and BOLD signal prediction.

### Hyperparameter settings

The input layer dimension ($N_{\text{in}}$) of the hypernetwork corresponded to the number of elements in the rsFCM, which was 99,235, representing the upper triangular elements of the correlation matrix between the 446 brain regions. As described in the “Preprocessing of sensory, task condition, and action data” section, there were 8 sensory input dimensions ($N_{\text{sensor}}$), 1 task condition dimension, 3 action output dimensions ($N_{\text{action}}$), and 20 BOLD output dimensions ($N_{\text{BOLD}}$). With these settings, the total number of learnable parameters in the main network ($N_{\text{params}\_\text{main}}$) is 173,623.

Through preliminary studies heuristically exploring a search space that included hidden unit sizes (50, 100, 300, 400, and 600 for the main network and 100, 200, and 400 for the hypernetwork), and dropout rates (0, 0.05, 0.01, and 0.3), we selected a hidden unit size of 400 for the main network, 200 for the hypernetwork, and a dropout rate of 0.1. Furthermore, the weighting coefficients for action and BOLD signals in the loss function ($\lambda_{\text{action}}$, $\lambda_{\text{BOLD}}$) were both set to 1. These preliminary explorations focused on identifying a configuration that ensured stable convergence. Regarding convergence criteria, we confirmed through visual inspection of learning curves in preliminary studies that validation loss showed a consistent downward trend and plateaued, indicating negligible further reduction and no signs of overfitting (Fig. S1).

To validate these architectural choices and the weighting coefficients, we conducted a post hoc systematic sensitivity analysis on a representative subset of the dataset (*N* = 50) trained for 300 epochs. First, we explored the search space for the main network’s hidden size (100, 200, 400, and 500), the hypernetwork’s hidden size (100, 200, and 300), and the dropout rate (0, 0.1, and 0.2). The results confirmed that the selected parameters ($N_{\text{hidden}\_\text{main}}$ = 400, $N_{\text{hidden}\_\text{hyper}}$ = 200, dropout = 0.1) offered the optimal balance between validation loss reduction, computational efficiency, and generalization (see Table S7 for detailed comparisons). Second, we evaluated the robustness of the weighting coefficients ($\lambda_{\text{action}}$, $\lambda_{\text{BOLD}}$) by comparing the balanced setting against action-focused and BOLD-focused schemes. This analysis supported the balanced setting ($\lambda_{\text{action}}$ = $\lambda_{\text{BOLD}}$ = 1) as essential for capturing both behavioral and neural dynamics simultaneously (Table S8).

The training data sequences were segmented into 262 time steps (20.96 s); this segmentation served as a data augmentation strategy to increase the effective number of training examples. We used a batch size of 50 and the Adam optimization algorithm with a learning rate of $10^{-6}$. To further ensure robust generalization despite the large number of generated parameters, we relied on the implicit regularization provided by the hypernetwork’s bottleneck architecture (generating weights from 200 latent units), combined with the explicit dropout (rate = 0.1). The maximum number of epochs was set to 800, and early stopping triggered by validation loss monitoring was used to prevent overfitting.

### GLM analysis

To quantitatively evaluate BOLD signal changes in response to task stimuli, we conducted a GLM analysis, a method commonly used in brain fMRI analysis [63]. To assess neural activity in response to stimuli, we used face and shape conditions as regressors for the Emotional Faces task and congruent and incongruent conditions as regressors for the Stroop task. For each participant’s BOLD signal data, task events were temporally locked to the start of each trial, and the Glover function was applied as a canonical hemodynamic response function (HRF). In addition, we included the first temporal derivative of the HRF in the regressors to capture temporal variations in the hemodynamic response. The design matrix of explanatory variables also incorporated 8 basis functions using the discrete cosine transform with a cutoff frequency of 0.01 Hz to account for potential low-frequency signal drift, along with a constant term representing the baseline. This enhances the model’s robustness against high-frequency noise.

Individual-level GLM analyses were conducted independently for each brain region using the least-squares method. The primary contrasts of interest were “face condition > shape condition” for the Emotional Faces task and “incongruent condition > congruent condition” for the Stroop task. Specifically, we evaluated contrasts representing differences in regression coefficients between conditions and calculated *t*-statistics by dividing the obtained contrast estimates by their standard errors to determine statistical significance. The *t*-statistics for the amygdala derived from these results were used in the model-based functional indicator for the treatment intervention simulation, as described later.

For group-level statistical inference, we conducted one-sample *t* tests (against 0) across all participants for each brain region, using *t*-statistics of contrasts obtained from individual-level GLM analyses. This approach enabled us to identify brain regions with significant group-level activity in response to the task stimuli. The results are shown in Fig. 3B.

### Assessment of functional indicators in the model

In this study, we evaluated the functional indicators related to emotional and cognitive processes, as reproduced within our model, by drawing a conceptual alignment with the RDoC framework. These assessments are based on the action and brain activity sequences generated by the main network during task performance. For the RDoC Negative Valence Systems domain, we quantified an indicator of affective response by measuring the strength of amygdala responses in BOLD signal sequences generated by the main network in response to negative facial expression stimuli during the Emotional Faces task. The amygdala plays a central role in the emotional response networks [50]. On the basis of the finding that the right amygdala is particularly involved in processing negative emotions [77], we used the response strength of the right amygdala, specifically, the *t*-statistic obtained from the individual-level GLM analysis mentioned earlier, as an indicator of negative affective response intensity in the model. We quantified an indicator for the RDoC Cognitive Systems domain, specifically processing speed, based on reaction times calculated from action sequences generated by the main network during Emotional Face and Stroop tasks. Reaction time is known to correlate strongly with a wide range of cognitive functions [78] and serves as a key measure of processing speed. Specifically, we calculated the time interval from the appearance of the visual stimuli to the occurrence of button-press actions, considering that each time step in the sequence was 80 ms.

### PLS analysis

In this study, we applied PLS analysis to examine the relationship between the functional indicators (affective response and processing speed) reproduced in the model and the weight parameters of the main network. When the vector for a specific functional indicator is denoted as s ($s\in {\mathbb{R}}^{N_{\text{participants}}}$), the objective of PLS is to identify latent variables in the weight parameters of the main network ($W_{\text{main}}\in {\mathbb{R}}^{N_{\text{participants}}\times N_{\text{params}\_\text{main}}}$) that maximize covariance with this indicator. This is equivalent to finding a coefficient vector v that satisfies the following condition. Prior to the PLS analysis, both the functional indicator vector s and weight parameter matrix $W_{\text{main}}$ were standardized.

$$v=\underset{\left\|u\right\|=1}{\text{argmax}}\left|\text{cov}\left(W_{\text{main}}u,s\right)\right|$$ (12)

Specifically, the coefficient vector v was calculated using the following formula:

$$v=\frac{W_{\text{main}}^{T}s}{\left\|W_{\text{main}}^{T}s\right\|}$$ (13)

The coefficient vector v indicates the direction in the weight–parameter space of the main network, and the score vector $z_{1}$ ($z_{1}$ = $W_{\text{main}}v$) obtained by projection onto this direction represents the first latent variable of $W_{\text{main}}$ that has the strongest association with the functional indicator s.

To identify the optimal intervention direction, specifically for affective response and processing speed, we conducted separate PLS regression analyses. One analysis used affective response data as a target functional indicator (s) and the other used processing speed data as s. This approach yielded 2 distinct coefficient vectors: $v_{\text{affective}}$ (the coefficient vector corresponding to the affective response, also called the affective vector) and $v_{\text{cognitive}}$ (the coefficient vector corresponding to processing speed, also called the cognitive vector). These vectors represent the optimal directions in the main network parameter space that most efficiently influence the changes in each functional indicator.

To validate the $v_{\text{affective}}$ and $v_{\text{cognitive}}$ vectors obtained through PLS analysis, we calculated the Pearson correlation coefficients between each vector and the corresponding functional indicator s across individuals. Strong negative correlations were confirmed for affective response (*r* = −0.59) and processing speed (*r* = −0.68). These results indicate that modifying weight parameters ($W_{\text{main}}$) in the direction specified by $v_{\text{affective}}$ leads to a weakened amygdala response to negative facial expressions (representing the target impact on affective response), and modifying them in the direction of $v_{\text{cognitive}}$ leads to decreased reaction time (representing the target impact on processing speed).

### Investigation of optimal manipulation for rsFCM

In this study, we utilized affective and cognitive vectors to examine manipulation strategies for rsFCM that efficiently affected affective responses and processing speed. These vectors represent the directions of change in the main network weight parameters ($W_{\text{main}}$) obtained from PLS analysis. To elucidate the changes in rsFCM that would shift $W_{\text{main}}$ along these vector directions, we applied the gradient-based visualization method SmoothGrad [79] to the hypernetwork. SmoothGrad evaluates the importance of input elements by calculating the gradient of the output with respect to each input element. This method overcomes the limitations of simple gradient methods, which are susceptible to noise, by adding small random noises to the input multiple times and averaging the gradients of the output for each noise-added input, thereby estimating robust importance values.

In our implementation, referencing the original SmoothGrad recommendations [79], we input the rsFCMs of all participants into the trained hypernetwork with a sampling frequency of 100 and a noise level of 0.1. To validate these choices, we performed a sensitivity analysis with varying sampling frequencies (50, 100, and 200) and noise levels (0.05, 0.1, 0.2, and 0.3). We confirmed that the resulting group-averaged gradient maps were highly robust, with Pearson correlation coefficients exceeding 0.99 across all comparisons (Table S9). Noise was generated following a normal distribution, with the SD calculated by multiplying the SD of the rsFCM by the noise level. Perturbed inputs were created by adding this noise to the input data. Each noise-added input was passed through the hypernetwork, and the outputs were *z*-score-normalized. We then obtained the first latent variable ($z_{1}$) corresponding to the cognitive and emotional vectors using PLS regression. The gradients of the input elements with respect to the value of the first latent variable were calculated and averaged across all sampling iterations to determine the contribution of the inputs using SmoothGrad. These averaged gradients indicate the degree of influence of each input element on the first latent variable, and serve as indicators for evaluating which elements of rsFCM are important for changing $W_{\text{main}}$ in the direction of the affective and cognitive vectors. In Fig. 4A to D, to show the general trend across all participants, we averaged the gradients obtained for rsFCM across the participants.

### Prediction of individual treatment effects based on simulation

We conducted a virtual treatment simulation to predict the individual treatment effects by modifying the weight parameters of each participant’s main network along the affective and cognitive vectors. The postmanipulation weight parameters of the main network for the ith participant, ${W^{\prime }}_{\text{main}}^{\left(i,:\right)}$, are represented by the following equation:

$${W^{\prime }}_{\text{main}}^{\left(i,:\right)}=W_{\text{main}}^{\left(i,:\right)}+\alpha v^{T}$$ (14)

where $W_{\text{main}}^{\left(i,:\right)}$ represents the premanipulation weight parameters of the main network. The scalar value α represents the intensity of the manipulation, which was set to twice the SD of the latent variable score $z_{1}$ in this study ($\alpha =2{\mathrm{SD}}_{z_{1}}$). We validated the appropriateness of this intensity through a sensitivity analysis, which confirmed a monotonic and clinically realistic dose–response relationship up to 2.5 SD (Fig. S7). Furthermore, to ensure that the linear manipulation did not lead to degenerate network behavior, we verified that task performance and action distributions were preserved after the intervention (Fig. S8). The vector v is either the affective vector or the cognitive vector, indicating the direction in the weight space of the main network that affects the affective response or processing speed.

Using the main network with postmanipulation weight parameters ${W^{\prime }}_{\text{main}}^{\left(i,:\right)}$ for each participant, we generated predictive action and BOLD signal time series in response to sensory stimuli in the Emotional Faces and Stroop tasks. From the predicted behavioral and neural activity time-series data, we calculated the postmanipulation affective response and processing speed in the model. Intervention effects were evaluated by comparing these values with the corresponding premanipulation values.

## Data Availability

The raw neuroimaging data and all behavioral measures used in the TCP dataset are accessible through OpenNeuro (https://openneuro.org/datasets/ds005237). The raw and processed neuroimaging data and all behavioral measures will be made publicly available through the NDA (https://nda.nih.gov/edit_collection.html?id=3552). The code is publicly available at https://osf.io/zuqf6/.
