# Supplementary material for: Digital Twin Brain: Generating Multitask Behavior from Connectomes for Personalized Therapy
Source: BME Front. 2026 Feb 12;7:0231. doi: 10.34133/bmef.0231 (PMC12895553; doi:10.34133/bmef.0231)
Supplement: Supplementary 1 — Methods S1 and S2 Figs. S1 to S8 Tables S1 to S9 [file bmef.0231.f1.docx]

Supplementary Materials for

**Digital Twin Brain: Generating Multitask Behavior from Connectomes
for Personalized Therapy**

Yuta Takahashi^1,2^*, Takafumi Soda^1^, Hiroaki Tomita^2^, Yuichi Yamashita^1^*

1. Department of Information Medicine, National Center of Neurology and Psychiatry, Tokyo, Japan

2. Department of Psychiatry, Graduate School of Medicine, Tohoku University, Sendai, Japan

***Correspondence**

Yuta Takahashi

Email: ytakaha@ncnp.go.jp

Yuichi Yamashita

Email: yamay@ncnp.go.jp

**Supplementary Methods 1:** **Cross-Participant Generalization Analysis**

To evaluate the model’s capability to generalize to novel individuals, we performed a split-half cross-validation analysis. This approach assesses whether the Hypernetwork can infer accurate Digital Twin Brain (DTB) parameters for participants strictly excluded from the training process.

1. Data Partitioning Strategy

For this generalization analysis, we adopted a participant-level split, distinct from the temporal split used in the main text.

1. First, for every participant, we utilized the full extent of the dataset without applying the temporal split (separating first and second halves) used in the main text.
2. We then randomly partitioned the participant cohort into two independent subsets of equal size:
   - Training Group ($N_{train}=50\%$): Data from these participants were used exclusively to train the Hypernetwork.
   - Held-out Testing Group ($N_{test}=50\%$): These participants were completely excluded from the training process and were used solely for evaluation.

This design ensures that the evaluation reflects the model's performance on strictly "unseen" individuals, utilizing their full data to test the robustness of the connectome-to-parameter mapping.

2. Training, Evaluation, and Metrics

The procedures for training the Hypernetwork, inferring DTB parameters from connectomes, and simulating behavioral and neural data followed the identical protocols described in the main text (see *Methods* section).

- Training & Inference: The Hypernetwork learned to map connectomes to DTB parameters using the Training Group. For the Testing Group, parameters were inferred solely from their connectomes without any individual calibration.
- Performance Metrics: We evaluated the model using the same three metrics defined in the main analysis: Action Choice Accuracy, Reaction Time (RT) Correlation, and BOLD Signal Similarity (pooled GLM t-values across brain regions).

**Supplementary Methods 2: Direct Input Model**

To empirically validate the theoretical advantage of the Hypernetwork's multiplicative weight modulation over an additive input strategy, we implemented and evaluated a "Direct Input" baseline model. This model represents a shared-weight architecture where connectome features act as a static bias to the recurrent units.

**1. Direct Input Model Architecture**

The Direct Input model utilizes the same Recurrent Neural Network (RNN) structure (e.g., hidden unit size, output layer) as the main network in the Hypernetwork architecture, but with fixed, shared weights ($W_{in}$, $W_{rec}$, $W_{out}$) across all participants.

Instead of generating the weights, the connectome information is integrated directly into the recurrent computation at every time step:

1. Connectome Feature Projection: The individual's connectome features ($c$) are first passed through a Multi-Layer Perceptron (MLP) with the same structure (number of layers and hidden units) as the Hypernetwork's encoder. This MLP outputs a 200-dimensional vector, which is then directly used as the connectome projection, $c_{proj}$. This 200-dimensional output matches the number of hidden units used in the MLP layers.
2. Additive Integration: The connectome projection ($c_{proj}$) is concatenated with the sensory input ($x_{t}$) before being weighted by $W_{in}$. The hidden state update rule ($h_{t}$) thus becomes:

$$h_{t}=\sigma\left( W_{in}x_{t}+W_{rec}h_{t-1}+W_{c}c_{proj}+b \right)$$

This formulation ensures that the connectome influences the circuit through a static, additive bias $c_{proj}$ (integrated as part of the input stream), rather than modulating the dynamics via multiplicative changes to the weights ($W_{rec}$ and $W_{in}$).

**2. Training and Evaluation**

The Direct Input model was trained on the entire cohort simultaneously, optimizing the single set of shared weights ($W_{in}$, $W_{rec}$, $W_{out}$, and the MLP weights) to minimize prediction error across all individuals and both tasks (Emotional Faces and Stroop).

- Objective: The shared parameters were optimized using the identical loss function as the main Hypernetwork model to achieve the highest mean accuracy and lowest loss across the population, attempting to capture the average relationship between input stimuli, connectome features, and output behavior.
- Evaluation: Performance was evaluated using the same within-individual approach as the main Hypernetwork analysis (temporal split: first half trained, second half tested).
- Metrics: We used the same three metrics: Action Prediction Accuracy, Reaction Time Correlation ($r$), and BOLD signal Prediction ($r$ of GLM t-values), for direct comparison with the Hypernetwork results (see Supplementary Table 5 and Supplementary Figure 8).

The low performance of this additive baseline (Supplementary Table 5) confirms that the complex inter-individual heterogeneity present in these neurobehavioral tasks cannot be sufficiently modeled by shared circuit dynamics supplemented only by a static additive bias.

**Supplementary Figure 1. Learning Curve.**

**
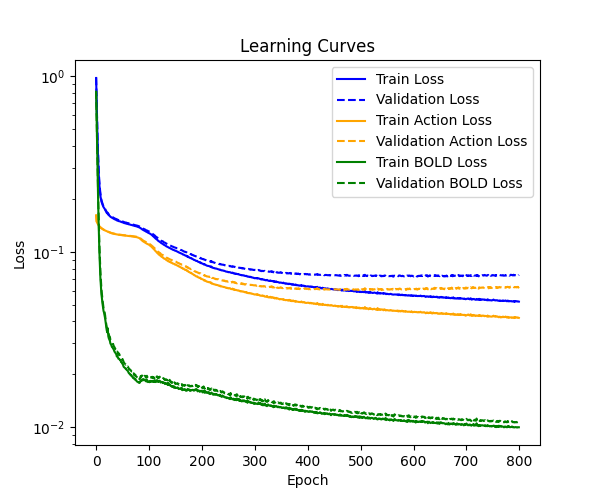
**

The learning curve shows the model performance for both the training (80%) and validation (20%) datasets split from the training dataset. The weight parameters of the model in the Hypernetwork were updated based on training loss. The model with the lowest validation loss was then selected for subsequent evaluation, using a separate test dataset.

**Supplementary Figure 2. Relationship between participant baseline accuracy and model-participant concordance rate.**

**
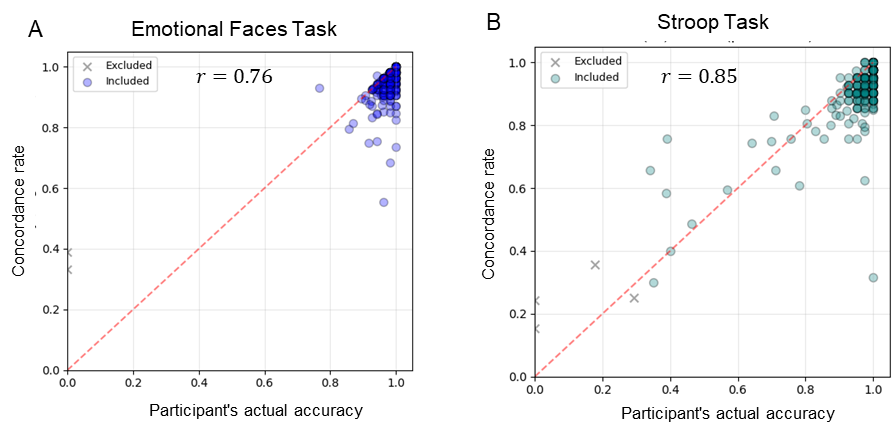
**

This figure displays the concordance rate (agreement between model predictions and participant choices) on the y-axis against the participant's actual accuracy on the x-axis. Participants with below-chance accuracy are marked with 'x'. We observed strong positive correlations (r=0.76 for Emotional Faces, r=0.85 for Stroop), indicating that modeling the specific choices of lower-performing participants is more challenging. Notably, data points are confined to the area at or below the diagonal line (the "empty upper triangle"). Since errors in these relatively simple cognitive tasks are often stochastic, it is theoretically impossible for a deterministic model to predict the exact timing of every random error. For instance, if a participant has 95% accuracy and the model also predicts with 95% accuracy, but errors occur randomly, the expected concordance (the sum of probabilities for both correct and both incorrect matches) is 0.95 $\times$0.95 + 0.05 $\times$0.05 $\approx$ 0.90, which is lower than the baseline accuracy. The observed distribution suggests that the model successfully learns the individual's systematic tendency to commit errors without overfitting to stochastic noise.

**Supplementary Figure 3. Comparison of model task accuracy versus participant actual accuracy.**

**
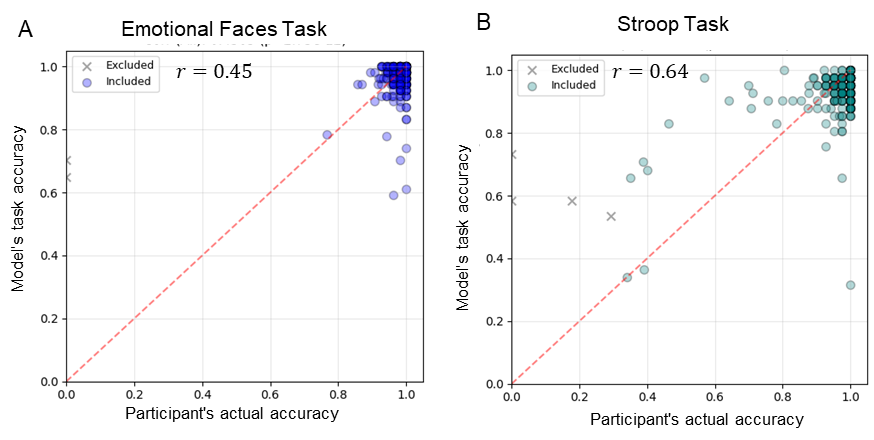
**

This figure compares the participant's actual task accuracy (x-axis) with the accuracy of the model's predicted actions (y-axis). Participants with below-chance accuracy are marked with 'x'. The model's predicted accuracy closely matched the participants' actual accuracy on average (Emotional Faces: 0.96 vs. 0.97; Stroop: 0.92 vs. 0.93), with moderate to strong correlations (r=0.45 and r=0.64, respectively). This demonstrates that the generated 'Digital Twin' is not an optimized artificial agent that solves the task perfectly, but rather a personalized model that faithfully replicates the individual's specific level of cognitive performance.

**Supplementary Figure 4. Learning curves comparing the Hypernetwork and Direct Input models.**

**
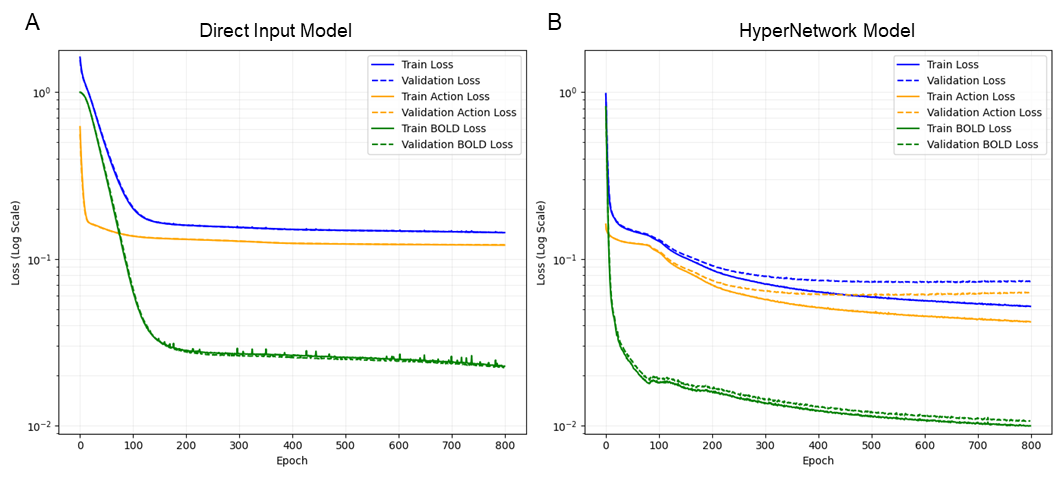
**

Validation loss trajectories during training for (A) the Direct Input model, where connectome features are added as a static bias to the recurrent layer, and (B) the proposed Hypernetwork model, where connectome features generate the weights of the recurrent layer. The x-axis represents training epochs, and the y-axis represents the training/validation loss. The Direct Input model (A) fails to significantly minimize the loss, indicating a limited capacity to capture complex individual behavioral patterns (consistent with the near-chance accuracy shown in Supplementary Table 5). In contrast, the Hypernetwork model (B) demonstrates rapid convergence and achieves a substantially lower loss, highlighting the superior efficiency and representational power of the multiplicative weight generation approach in modeling personalized dynamics.

**Supplementary Figure 5. Relationship Between Intervention Effects Across Different Functional Indicators**

**
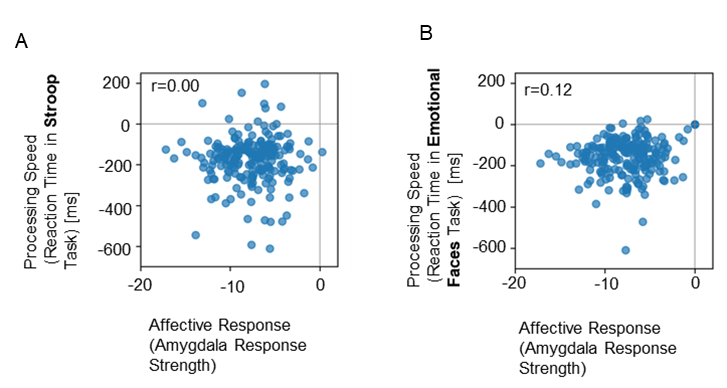
**

In both scatter plots, the x-axis represents the change in amygdala BOLD signal t-values (GLM) induced by the affective response intervention. The y-axis indicates the change in reaction times for the Stroop task in (A) and the emotional faces task in (B). In essence, these plots examine the relationship between the effect sizes of these paired indices, which were presented in Fig. 5D, E, and F.

**Supplementary Figure 6. Robustness of GLM t-statistic prediction against interpolation methods.**

**
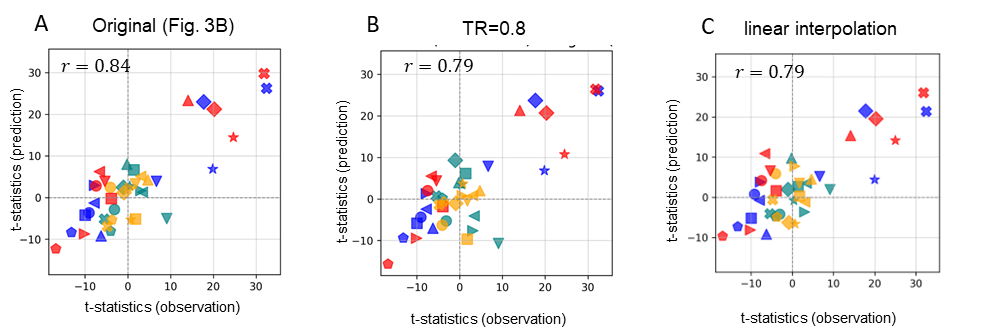
**

This figure compares the correlation between observed and predicted GLM t-statistics under different interpolation and evaluation schemes. (A) The original result from Figure 3B in the manuscript, derived using the standard cubic interpolation method. (B) Results evaluated using only the original fMRI acquisition time points (TR = 0.8 s), discarding all intermediate interpolated frames during the GLM analysis. (C) Results from a model retrained entirely using linear interpolation instead of cubic interpolation. Each panel displays a scatterplot comparing the observed and predicted GLM t-statistics for the Emotional Faces task. The correlation coefficients remain consistently high across all conditions (r = 0.84, 0.79, and 0.79, respectively), and the regional distributions are qualitatively similar. These results confirm that the high prediction performance is not an artifact of the cubic interpolation or artificial temporal smoothing, but rather reflects the model's ability to capture true task-induced neural dynamics.

**Supplementary Figure 7. Sensitivity analysis of simulated treatment effects across manipulation intensities.**

**
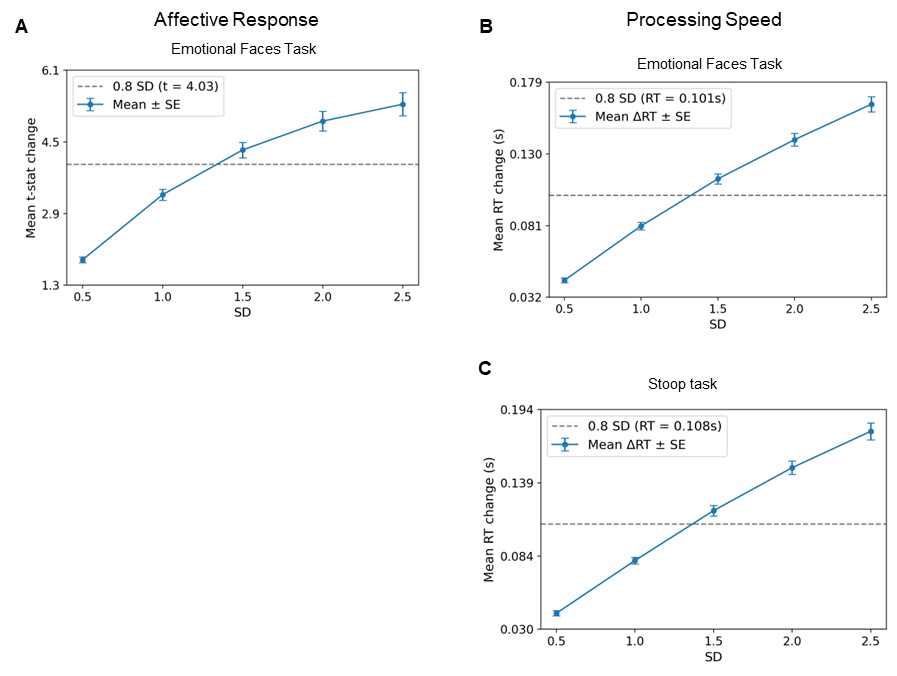
**

This figure illustrates the dose-response relationship between the manipulation intensity (α, ranging from 0.5 to 2.5 SD) and the magnitude of simulated treatment outcomes. (A) Change in amygdala BOLD signal GLM t-values (y-axis) as a function of manipulation intensity α (x-axis) during interventions targeting the Affective Response. The plot shows a monotonic reduction in amygdala activation as the intervention intensity increases. (B, C) Change in reaction times (y-axis) as a function of manipulation intensity α (x-axis) during interventions targeting Processing Speed. Panels (B) and (C) correspond to the results for the Emotional Faces task and Stroop task, respectively. Both panels demonstrate a monotonic decrease in reaction times (improved processing speed) with increasing α. In all panels, the dashed horizontal lines indicate the threshold for a large effect size (Cohen's d = 0.8). The results confirm that the chosen parameter for the main analysis (α = 2 SD) generates treatment effects that are quantitatively comparable to large clinical effects observed in empirical studies, while the monotonic trends suggest the model operates within a stable regime.

**Supplementary Figure 8. Verification of functional stability and local linearity under latent parameter manipulation.**

**
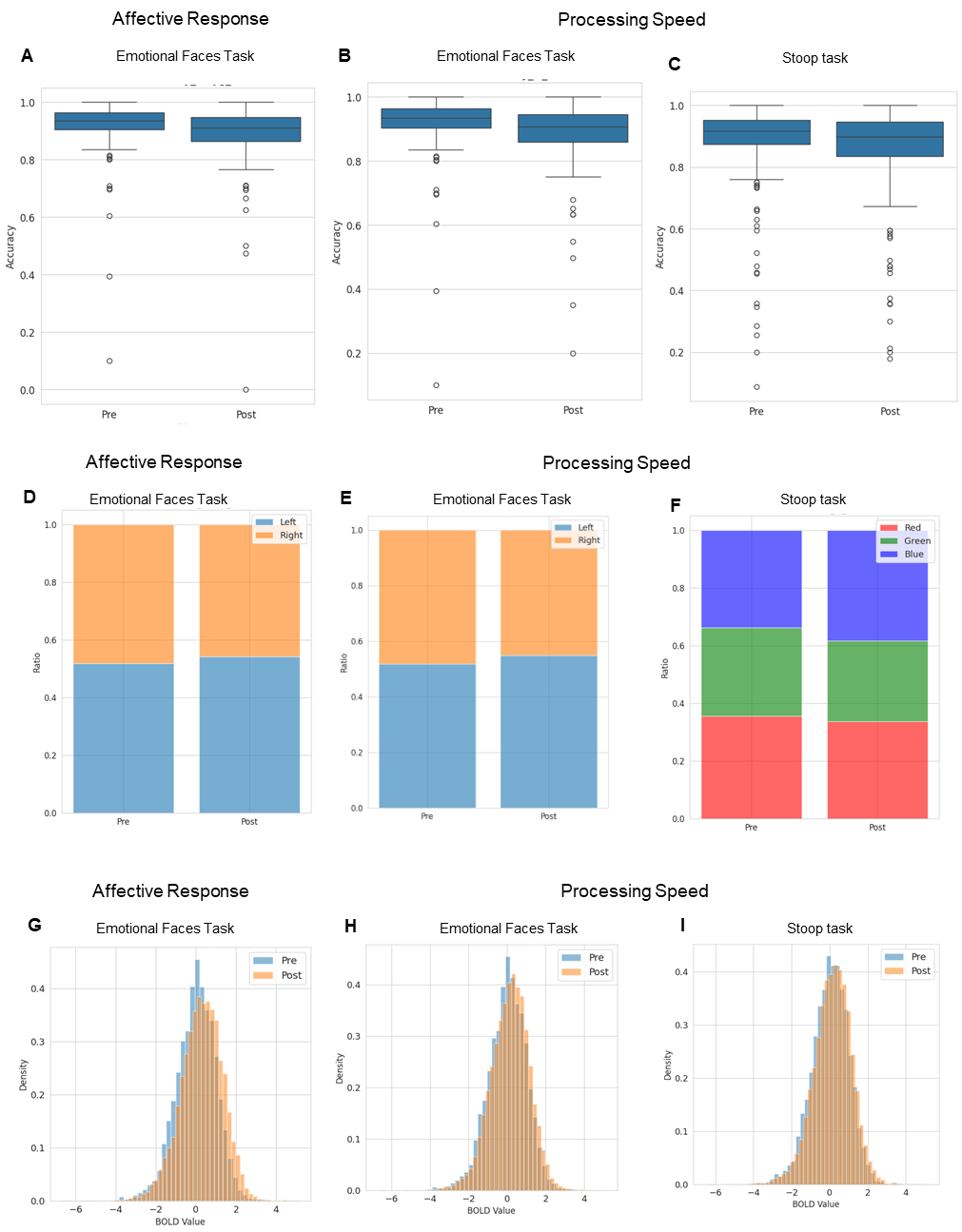
**

This figure demonstrates that the linear manipulation of model parameters (simulated intervention) does not lead to degenerate network behaviors, ensuring the model operates within a stable physiological regime.

(A–C) Preservation of Task Performance: Comparison of action prediction accuracy before (x-axis) and after (y-axis) intervention for Emotional Faces and Stroop tasks. Data points cluster along the diagonal, indicating that the core computational capabilities and high task performance are maintained despite the parameter perturbation.

(D–F) Prevention of Behavioral Collapse: Bar plots showing the ratio of action choices (e.g., Left vs. Right; Color responses) before and after intervention. The distributions remain largely unchanged, proving that the model retains appropriate decision-making diversity and has not exhibited a collapse of output diversity into trivial, repetitive patterns.

(G–I) Physiological Stability of Neural Dynamics: Histograms of predicted BOLD signals (GLM t-values) across all regions. The distributions after intervention (orange) overlap well with the original distributions (blue) and remain within the physiological range, showing no signs of signal explosion (saturation) or vanishing.

**Supplementary Table 1. All Clinical Measures and Their Correlation Coefficients with Cognitive (**$\boldsymbol{v}_{\mathbf{cognitive}}$**) and Affective (**$\boldsymbol{v}_{\mathbf{affective}}$**) Vectors.**

| Psychiatric Symptom Scale | Specific Scores | $r$ with $v_{\mathrm{cognitive}}$ | $r$ with $v_{\mathrm{affective}}$ |
| --- | --- | --- | --- |
| Anxiety Sensitivity Index | Total Score | 0.07 | -0.12 |
| Broad Autism Phenotype Questionnaire | Aloofness | 0.00 | 0.05 |
| Broad Autism Phenotype Questionnaire | Pragmatic Language | 0.02 | -0.01 |
| Broad Autism Phenotype Questionnaire | Rigidity | -0.16 | -0.04 |
| Broad Autism Phenotype Questionnaire | Total Score | -0.04 | 0.00 |
| Barratt Impulsiveness Scale | Total Score | -0.01 | -0.01 |
| Barratt Impulsiveness Scale | Attentional Impulsiveness | 0.05 | -0.05 |
| Barratt Impulsiveness Scale | Motor Impurlsiveness | -0.04 | 0.13 |
| Barratt Impulsiveness Scale | Non-planning Impulsiveness | -0.05 | 0.00 |
| Behavioral Inhibition/Activation Scale | Total Score | 0.13 | -0.13 |
| Behavioral Inhibition/Activation Scale | BAS Drive | -0.13 | 0.04 |
| Behavioral Inhibition/Activation Scale | BAS Fun Seeking | 0.01 | 0.06 |
| Behavioral Inhibition/Activation Scale | BAS Reward Responsiveness | 0.07 | -0.04 |
| Cognitive Emotion Regulation Questionnaire | Self-blame | 0.00 | 0.10 |
| Cognitive Emotion Regulation Questionnaire | Acceptance | 0.18 | 0.10 |
| Cognitive Emotion Regulation Questionnaire | Rumination | -0.04 | -0.04 |
| Cognitive Emotion Regulation Questionnaire | Positive Refocusing | 0.08 | 0.01 |
| Cognitive Emotion Regulation Questionnaire | Refocus on Planning | 0.02 | -0.07 |
| Cognitive Emotion Regulation Questionnaire | Positive Reappraisal | -0.08 | 0.09 |
| Cognitive Emotion Regulation Questionnaire | Putting into Perspective | 0.04 | -0.01 |
| Cognitive Emotion Regulation Questionnaire | Catastrophizing | 0.01 | 0.02 |
| Cognitive Emotion Regulation Questionnaire | Other-blame | 0.04 | -0.02 |
| Cognitive Failures Questionnaire | Total Score | 0.05 | 0.02 |
| Childhood Trauma Questionnaire | Emotional Abuse | -0.13 | 0.06 |
| Childhood Trauma Questionnaire | Emotional Neglect | -0.18 | 0.03 |
| Childhood Trauma Questionnaire | Physical Abuse | -0.19 | 0.05 |
| Childhood Trauma Questionnaire | Physical Neglect | -0.13 | 0.06 |
| Childhood Trauma Questionnaire | Sexual Abuse | -0.16 | 0.01 |
| Childhood Trauma Questionnaire | Validity Scale | 0.08 | 0.03 |
| Depression Anxiety Stress Scale | Depression Score | 0.01 | -0.06 |
| Depression Anxiety Stress Scale | Anxiety Score | -0.05 | -0.07 |
| Depression Anxiety Stress Scale | Stress Score | -0.02 | -0.07 |
| Domain-Specific Risk Taking | Ethical Risk Taking | 0.11 | 0.07 |
| Domain-Specific Risk Taking | Financial Risk Taking | 0.11 | 0.07 |
| Domain-Specific Risk Taking | Health/Safety Risk Taking | 0.14 | 0.11 |
| Domain-Specific Risk Taking | Recreational Risk Taking | 0.20 | 0.06 |
| Domain-Specific Risk Taking | Social Risk Taking | 0.02 | -0.01 |
| Domain-Specific Risk Taking | Social Risk Perception | -0.03 | -0.09 |
| Domain-Specific Risk Taking | Ethical Risk Perception | -0.20 | -0.08 |
| Domain-Specific Risk Taking | Financial Risk Perception | -0.08 | -0.18 |
| Domain-Specific Risk Taking | Health/Safety Risk Perception | -0.15 | -0.10 |
| Domain-Specific Risk Taking | Recreational Risk Perception | -0.18 | -0.07 |
| Domain-Specific Risk Taking | Overall Risk-Taking Score | 0.18 | 0.08 |
| Domain-Specific Risk Taking | Overall Risk Perception Score | -0.16 | -0.13 |
| Experiences in Close Relationships Inventory | Attachment Anxiety | -0.08 | 0.05 |
| Experiences in Close Relationships Inventory | Attachment Avoidance | 0.04 | 0.08 |
| Fagerstrom Test for Nicotine Dependence | Total Score | -0.08 | -0.09 |
| Multidimensional Scale for Perceived Social Support | Family Support | 0.06 | -0.12 |
| Multidimensional Scale for Perceived Social Support | Friends Support | 0.08 | -0.08 |
| Multidimensional Scale for Perceived Social Support | Significant Other Support | 0.01 | -0.13 |
| Multidimensional Scale for Perceived Social Support | Total Perceived Social Support | 0.06 | -0.13 |
| NEO Five Factor Inventory | Agreeableness | 0.05 | -0.14 |
| NEO Five Factor Inventory | Conscientiousness | -0.03 | -0.08 |
| NEO Five Factor Inventory | Extraversion | 0.03 | -0.09 |
| NEO Five Factor Inventory | Neuroticism | 0.03 | -0.07 |
| NEO Five Factor Inventory | Openness to Experience | 0.06 | 0.02 |
| Profile of Mood States | Tension sub-score | 0.06 | -0.11 |
| Profile of Mood States | Depression sub-score | 0.02 | -0.02 |
| Profile of Mood States | Anger sub-score | -0.03 | 0.03 |
| Profile of Mood States | Fatigue sub-score | 0.06 | -0.04 |
| Profile of Mood States | Confusion sub-score | -0.02 | -0.06 |
| Profile of Mood States | Vigour sub-score | 0.06 | 0.03 |
| Profile of Mood States | Total Score | 0.01 | -0.05 |
| Perceived Stress Scale | Total Score | -0.04 | 0.00 |
| Positive Urgency Measure | Total Score | 0.00 | 0.02 |
| Quick Inventory of Depressive Symptomatology | Total Score | 0.02 | -0.07 |
| Ruminative Responses Scale | Total Score | 0.07 | 0.02 |
| Retrospective Self-Report of Inhibition | Total Average Score | -0.07 | -0.01 |
| Retrospective Self-Report of Inhibition | Social/Situational Inhibition Score | -0.05 | 0.07 |
| Retrospective Self-Report of Inhibition | Fear/Illness Inhibition Score | 0.00 | -0.09 |
| Snaith-Hamilton Pleasure Scale | Total Score | -0.08 | 0.01 |
| Shipley Institute of Living Scale | Vocabulary Score | 0.07 | -0.04 |
| Shipley Institute of Living Scale | IQ Score | 0.17 | -0.03 |
| State-Trait Anxiety Inventory | State Anxiety | -0.04 | -0.04 |
| State-Trait Anxiety Inventory | Trait Anxiety | -0.01 | -0.02 |
| Temperament Character Inventory | Novelty Seeking | -0.08 | 0.05 |
| Temperament Character Inventory | Harm Avoidance | 0.08 | -0.05 |
| Temperament Character Inventory | Reward Dependence | 0.03 | -0.20 |
| Temperament Character Inventory | Persistence | -0.07 | -0.09 |
| Temperament Character Inventory | Self-Directedness | -0.06 | -0.11 |
| Temperament Character Inventory | Cooperativeness | 0.11 | -0.10 |
| Temperament Character Inventory | Self-Transcendence | -0.19 | 0.03 |
| Temperament Character Inventory | Total Score | -0.03 | -0.18 |
| Temporal Experience of Pleasure Scale | Anticipatory Pleasure | 0.10 | -0.06 |
| Temporal Experience of Pleasure Scale | Consummatory Pleasure | 0.10 | -0.18 |
| Test My Brain | Digit Symbol Matching Score | 0.42 | -0.04 |
| Test My Brain | gradCPT Score | -0.03 | 0.06 |
| Test My Brain | Memory Score | 0.05 | -0.15 |
| Word Sentence Association Paradigm | Word Association Score | -0.07 | -0.03 |
| Young Mania Rating Scale | Total Score | -0.09 | -0.03 |
| Columbia Suicide Severity Rating Scale | Passive Death Wish | -0.07 | -0.04 |
| Columbia Suicide Severity Rating Scale | Active Suicidal Ideation | -0.05 | -0.11 |
| Columbia Suicide Severity Rating Scale | Actual Suicide Attempt | -0.20 | 0.00 |
| Columbia Suicide Severity Rating Scale | Non-Suicidal Self-Injury | 0.06 | -0.15 |
| Columbia Suicide Severity Rating Scale | Interrupted Suicide Attempt | -0.20 | -0.10 |
| Columbia Suicide Severity Rating Scale | Aborted Suicide Attempt | -0.04 | 0.12 |
| Columbia Suicide Severity Rating Scale | Preparatory Suicidal Acts | -0.07 | -0.04 |
| Columbia Suicide Severity Rating Scale | Aggregate Suicidal Behavior | 0.12 | 0.04 |
| Clinical Global Impression | Severity of Illness | -0.17 | -0.12 |
| LIFE_RIFT Scale / Range of Impaired Functioning Tool | Total Score | -0.27 | 0.00 |
| Montgomery-Asberg Depression Rating Scale | Total Score | -0.14 | -0.09 |
| Multinomah Community Ability Scale | Total Score | 0.21 | 0.05 |
| Positive and Negative Syndrome Scale | General Psychopathology Score | -0.14 | -0.03 |
| Positive and Negative Syndrome Scale | Negative Symptoms Score | -0.10 | 0.05 |
| Positive and Negative Syndrome Scale | Positive Symptoms Score | -0.21 | -0.04 |
| Panic Disorder Severity Scale | Frequency of panic attacks | -0.03 | -0.24 |
| Psychiatric Disorder Status | Diagnostic Status | -0.06 | -0.04 |

**Supplementary Table 2. Top 10 functional connections identified by gradient analysis for modulating amygdala reactivity (Affective Response)**

| Direction | Rank | ROI1 | ROI2 | Area1 | Area2 | Hemi1 | Hemi2 | Raw Gradient | Z score |
| --- | --- | --- | --- | --- | --- | --- | --- | --- | --- |
| Strengthen | 1 | 17Networks_LH_DorsAttnB_PostC_6 | 17Networks_RH_SomMotB_S2_1 | Motor | Motor | LH | RH | 1.47 | 4.45 |
| Strengthen | 2 | 17Networks_RH_DefaultA_PFCd_2 | NAc-shell-lh | Prefrontal | Subcortical | RH | LH | 1.40 | 4.24 |
| Strengthen | 3 | 17Networks_LH_SalVentAttnA_Ins_1 | 17Networks_LH_ContA_PFCl_3 | Insula | Prefrontal | LH | LH | 1.38 | 4.16 |
| Strengthen | 4 | 17Networks_LH_SalVentAttnB_Ins_2 | 17Networks_LH_DefaultA_PFCm_2 | Insula | Prefrontal | LH | LH | 1.32 | 4.00 |
| Strengthen | 5 | 17Networks_LH_SomMotA_3 | 17Networks_LH_TempPar_2 | Motor | Temporal | LH | LH | 1.30 | 3.94 |
| Strengthen | 6 | 17Networks_LH_SalVentAttnA_ParOper_1 | 17Networks_RH_LimbicB_OFC_1 | Parietal | Prefrontal | LH | RH | 1.27 | 3.83 |
| Strengthen | 7 | 17Networks_LH_SomMotB_S2_3 | 17Networks_LH_ContB_PFClv_3 | Motor | Prefrontal | LH | LH | 1.25 | 3.79 |
| Strengthen | 8 | 17Networks_RH_ContA_IPS_1 | 17Networks_RH_DefaultA_PFCm_6 | Parietal | Prefrontal | RH | RH | 1.23 | 3.70 |
| Strengthen | 9 | 17Networks_LH_SomMotB_S2_5 | 17Networks_LH_DefaultA_pCunPCC_6 | Motor | Parietal | LH | LH | 1.21 | 3.67 |
| Strengthen | 10 | 17Networks_LH_LimbicB_OFC_5 | 17Networks_LH_DefaultB_Temp_3 | Prefrontal | Temporal | LH | LH | 1.21 | 3.67 |
| Weaken | 1 | 17Networks_LH_ContC_Cingp_1 | 17Networks_RH_ContA_IPS_3 | Limbic | Parietal | LH | RH | -1.43 | -4.32 |
| Weaken | 2 | 17Networks_LH_DorsAttnA_TempOcc_3 | aGP-lh | Temporal | Subcortical | LH | LH | -1.39 | -4.20 |
| Weaken | 3 | 17Networks_LH_DefaultB_IPL_1 | buckner_crblm_7Networks_3-lh | Parietal | Cerebellum | LH | LH | -1.28 | -3.89 |
| Weaken | 4 | 17Networks_RH_LimbicB_OFC_1 | 17Networks_RH_DefaultB_PFCd_2 | Prefrontal | Prefrontal | RH | RH | -1.27 | -3.85 |
| Weaken | 5 | 17Networks_LH_LimbicA_TempPole_6 | pGP-rh | Temporal | Subcortical | LH | RH | -1.26 | -3.81 |
| Weaken | 6 | 17Networks_RH_DorsAttnB_TempOcc_1 | 17Networks_RH_DorsAttnB_FEF_1 | Temporal | Motor | RH | RH | -1.22 | -3.70 |
| Weaken | 7 | 17Networks_LH_TempPar_1 | pPUT-lh | Temporal | Subcortical | LH | LH | -1.22 | -3.69 |
| Weaken | 8 | 17Networks_RH_DorsAttnA_SPL_6 | THA-VP-lh | Parietal | Subcortical | RH | LH | -1.21 | -3.68 |
| Weaken | 9 | 17Networks_LH_DefaultA_PFCd_2 | 17Networks_LH_DefaultA_pCunPCC_5 | Prefrontal | Parietal | LH | LH | -1.21 | -3.68 |
| Weaken | 10 | 17Networks_LH_DorsAttnB_PrCv_1 | 17Networks_LH_DefaultB_PFCv_4 | Motor | Prefrontal | LH | LH | -1.21 | -3.66 |

**Supplementary Table 3. Top 10 functional connections identified by gradient analysis for improving processing speed (Cognitive Control)**

| Direction | Rank | ROI1 | ROI2 | Area1 | Area2 | Hemi1 | Hemi2 | Raw Gradient | Z score |
| --- | --- | --- | --- | --- | --- | --- | --- | --- | --- |
| Strengthen | 1 | 17Networks_RH_LimbicB_OFC_1 | 17Networks_RH_DefaultB_PFCd_2 | Prefrontal | Prefrontal | RH | RH | 1.44 | 4.31 |
| Strengthen | 2 | 17Networks_LH_DorsAttnA_TempOcc_3 | aGP-lh | Temporal | Subcortical | LH | LH | 1.42 | 4.24 |
| Strengthen | 3 | 17Networks_LH_ContC_Cingp_1 | 17Networks_RH_ContA_IPS_3 | Limbic | Parietal | LH | RH | 1.39 | 4.16 |
| Strengthen | 4 | 17Networks_RH_ContC_pCun_3 | aGP-rh | Parietal | Subcortical | RH | RH | 1.36 | 4.07 |
| Strengthen | 5 | 17Networks_LH_LimbicA_TempPole_6 | pGP-rh | Temporal | Subcortical | LH | RH | 1.29 | 3.86 |
| Strengthen | 6 | 17Networks_LH_DefaultB_IPL_1 | buckner_crblm_7Networks_3-lh | Parietal | Cerebellum | LH | LH | 1.25 | 3.75 |
| Strengthen | 7 | 17Networks_RH_ContB_PFClv_1 | 17Networks_RH_DefaultC_IPL_2 | Prefrontal | Parietal | RH | RH | 1.21 | 3.62 |
| Strengthen | 8 | 17Networks_RH_DorsAttnB_TempOcc_1 | 17Networks_RH_DorsAttnB_FEF_1 | Temporal | Motor | RH | RH | 1.20 | 3.57 |
| Strengthen | 9 | 17Networks_LH_SomMotB_S2_2 | 17Networks_RH_SomMotA_12 | Motor | Motor | LH | RH | 1.19 | 3.57 |
| Strengthen | 10 | 17Networks_RH_DorsAttnB_TempOcc_1 | 17Networks_RH_DorsAttnB_PostC_1 | Temporal | Motor | RH | RH | 1.19 | 3.55 |
| Weaken | 1 | 17Networks_LH_DorsAttnB_PostC_6 | 17Networks_RH_SomMotB_S2_1 | Motor | Motor | LH | RH | -1.39 | -4.15 |
| Weaken | 2 | 17Networks_LH_SalVentAttnA_Ins_1 | 17Networks_LH_ContA_PFCl_3 | Insula | Prefrontal | LH | LH | -1.39 | -4.12 |
| Weaken | 3 | 17Networks_RH_SalVentAttnA_FrMed_3 | THA-VP-lh | Prefrontal | Subcortical | RH | LH | -1.37 | -4.06 |
| Weaken | 4 | 17Networks_LH_DefaultC_PHC_1 | 17Networks_RH_ContA_IPS_2 | Limbic | Parietal | LH | RH | -1.35 | -4.01 |
| Weaken | 5 | 17Networks_RH_DefaultA_PFCd_2 | NAc-shell-lh | Prefrontal | Subcortical | RH | LH | -1.33 | -3.95 |
| Weaken | 6 | 17Networks_LH_ContA_PFClv_1 | 17Networks_RH_ContB_Temp_1 | Prefrontal | Temporal | LH | RH | -1.31 | -3.89 |
| Weaken | 7 | 17Networks_LH_SomMotB_S2_5 | 17Networks_LH_DefaultA_pCunPCC_6 | Motor | Parietal | LH | LH | -1.28 | -3.82 |
| Weaken | 8 | 17Networks_LH_DefaultB_PFCv_1 | 17Networks_RH_VisPeri_StriCal_1 | Prefrontal | Occipital | LH | RH | -1.28 | -3.81 |
| Weaken | 9 | 17Networks_RH_ContA_IPS_1 | 17Networks_RH_DefaultA_PFCm_6 | Parietal | Prefrontal | RH | RH | -1.28 | -3.79 |
| Weaken | 10 | 17Networks_LH_VisCent_ExStr_6 | 17Networks_RH_SalVentAttnA_FrMed_3 | Occipital | Prefrontal | LH | RH | -1.27 | -3.78 |

**Supplementary Table 4. Comparison of Generalization Performance (Within-individual vs. Cross-participant).**

|  | Task | Within-individual (Reported in Main Text) | Cross-Participant |
| --- | --- | --- | --- |
| Action Choice Accuracy | Emotion | 0.94 ±0.06 | 0.93 ±0.09 |
|  | Stroop | 0.90 ±0.10 | 0.87 ±0.15 |
| Reaction Time Correlation (r) | Emotion | 0.90 [0.87, 0.92] | 0.42 [0.25, 0.57] |
|  | Stroop | 0.85 [0.80, 0.88] | 0.34 [0.17, 0.50] |
| BOLD Signal Correlation (GLM t-values, r) | Pooled | 0.84 [0.72, 0.91] | 0.83 [0.71, 0.91] |

Description of Results:

To investigate the cross-participant generalization of our framework, we conducted a split-half validation where the model was trained on 50% of the participants and tested on the remaining 50% (held-out individuals). In the table above, accuracy values represent Mean ± SD across participants, and correlation values represent Pearson’s r with 95% Confidence Intervals.

The results show that while action choices and BOLD signal patterns generalize robustly to new individuals (r = 0.83), reaction time (RT) predictions exhibit a performance decrease (r $\approx$ 0.34-0.42). This discrepancy likely reflects the higher inter-individual variability in reaction time compared to task-related choices and regional hemodynamic responses.

**Supplementary Table 5. Comparison of predictive performance between the proposed Hypernetwork model and the Direct Input baseline model.**

| **Metric** |  | **Hypernetwork** | **Direct Input** |
| --- | --- | --- | --- |
| **Action Prediction Accuracy** | Emotion | 0.94 ±0.06 | 0.50 ±0.07 |
|  | Stroop | 0.90 ±0.10 | 0.29 ±0.06 |
| **Reaction Time Correlation (r)** | Emotion | 0.90 [0.87, 0.92] | -0.03 [-0.16, 0.10] |
|  | Stroop | 0.85 [0.80, 0.88] | -0.03 [-0.17, 0.10] |
| **Neural Prediction (GLM t-values, r)** | Pooled | 0.84 [0.72, 0.91] | 0.68 [0.47, 0.82] |

Description of Results:

To validate the necessity of the Hypernetwork architecture, we performed an ablation study comparing it with a "Direct Input" model. In this baseline, connectome features were concatenated with sensory inputs and fed into a standard RNN with shared weights (Supplementary Methods 2). In the table above, accuracy values represent Mean ± SD across participants, and correlation values represent Pearson’s r with 95% Confidence Intervals.

The results demonstrate that the Direct Input model fails to capture individual behavioral variability, with action accuracies near chance levels and reaction time correlations that did not differ significantly from zero (95% CI includes 0). Although the BOLD signal prediction was partially maintained, the overall results suggest that multiplicative modulation of weights via the Hypernetwork is essential for integrating connectome information into the functional dynamics of the model.

**Supplementary Table 6**

**Region Names of Brain Areas Used in the Current Study From the Task fMRI Data.**

| Hemisphere | Brain Region | ROI Name in TCP dataset |
| --- | --- | --- |
| Left | Anterior Prefrontal Cortex | 17Networks_LH_DefaultA_PFCm_5 |
| Left | Ventrolateral Prefrontal Cortex | 17Networks_LH_ContA_PFCl_2 |
| Left | Premotor Cortex | 17Networks_LH_ContB_PFCd_1 |
| Left | Primary Motor Cortex | 17Networks_LH_SomMotA_16 |
| Left | Primary Sensory Cortex | 17Networks_LH_SomMotA_4 |
| Left | Superior Parietal Cortex | 17Networks_LH_DorsAttnA_SPL_2 |
| Left | Auditory Cortex | 17Networks_LH_SomMotB_Aud_2 |
| Left | Higher Visual Cortex | 17Networks_LH_DorsAttnA_TempOcc_1 |
| Left | Primary Visual Cortex | 17Networks_LH_VisCent_ExStr_5 |
| Left | Amygdala | mAMY-lh |
| Right | Anterior Prefrontal Cortex | 17Networks_RH_DefaultA_PFCm_5 |
| Right | Ventrolateral Prefrontal Cortex | 17Networks_RH_ContA_PFCl_2 |
| Right | Premotor Cortex | 17Networks_RH_ContA_PFCd_1 |
| Right | Primary Motor Cortex | 17Networks_RH_SomMotA_17 |
| Right | Primary Sensory Cortex | 17Networks_RH_SomMotA_4 |
| Right | Superior Parietal Cortex | 17Networks_RH_DorsAttnA_SPL_3 |
| Right | Auditory Cortex | 17Networks_RH_SomMotB_Aud_3 |
| Right | Higher Visual Cortex | 17Networks_RH_DorsAttnA_TempOcc_1 |
| Right | Primary Visual Cortex | 17Networks_RH_VisCent_ExStr_6 |
| Right | Amygdala | mAMY-rh |

BOLD signals generated by the Main Network were trained using the aforementioned brain regions. Note that all 446 brain regions were used to construct the resting functional connectivity matrix (rsFCM) input to the Hyper Network (see methods for details). The ROI represents the Region of Interest.

**Supplementary Table 7. Hyperparameter Sensitivity Analysis.**

**A. Main Network Hidden Size**

| **Configuration (Main Net Hidden)** | **Val Loss** | **Action Loss** | **BOLD Loss** |
| --- | --- | --- | --- |
| hidden_size=500 | 0.112240 | 0.094825 | 0.017425 |
| **hidden_size=400 (Selected)** | **0.112290** | **0.095425** | **0.016875** |
| hidden_size=200 | 0.130856 | 0.118275 | 0.012575 |
| hidden_size=100 | 0.131669 | 0.120300 | 0.011350 |

**B. Hypernetwork Hidden Size**

| **Configuration (Hypernet Hidden)** | **Val Loss** | **Action Loss** | **BOLD Loss** |
| --- | --- | --- | --- |
| Hypernetwork hidden size: 100 | 0.142947 | 0.124875 | 0.018075 |
| **Hypernetwork hidden size: 200 (Selected)** | **0.112290** | **0.095425** | **0.016875** |
| Hypernetwork hidden size: 300 | 0.094866 | 0.080525 | 0.014325 |

**C. Dropout Rate**

| **Configuration (Dropout)** | **Val Loss** | **Action Loss** | **BOLD Loss** |
| --- | --- | --- | --- |
| Dropout rate: 0 | 0.108017 | 0.090650 | 0.017325 |
| **Dropout rate: 0.1 (Selected)** | **0.112290** | **0.095425** | **0.016875** |
| Dropout rate: 0.2 | 0.120640 | 0.103975 | 0.016725 |

To justify the hyperparameter selection, we conducted a sensitivity analysis using a subset of 50 participants trained for 300 epochs. The tables below show the validation loss (total), action loss, and BOLD loss for each configuration.

Note: For the final model, we selected a Main network hidden size of 400 and a Hypernetwork hidden size of 200 to balance performance and computational cost. A dropout rate of 0.1 was selected to ensure robust BOLD signal prediction and regularization.

**Supplementary Table 8. Sensitivity Analysis of Loss Function Weights**

| **Metric** | **Action-focused  (**$\boldsymbol{\lambda}_{\boldsymbol{action}}$**= 10,** $\boldsymbol{\lambda}_{\boldsymbol{BOLD}}$ **= 1)** | **Balanced (**$\boldsymbol{\lambda}_{\boldsymbol{action}}$**=1,**$\boldsymbol{\lambda}_{\boldsymbol{BOLD}}$**= 1)** | **BOLD-focused (**$\boldsymbol{\lambda}_{\boldsymbol{action}}$**= 1,** $\boldsymbol{\lambda}_{\boldsymbol{BOLD}}$ **= 10)** |
| --- | --- | --- | --- |
| Action Accuracy (Emotion) | 0.78 ±0.05 | 0.62 ±0.09 | 0.51 ±0.07 |
| RT Correlation (Emotion) | 0.77 [0.61, 0.87] | 0.83 [0.71, 0.90] | -0.15 [-0.42, 0.15] |
| Action Accuracy (Stroop) | 0.91 ±0.04 | 0.39 ±0.11 | 0.38 ±0.12 |
| RT Correlation (Stroop) | 0.84 [0.74,0 .91] | 0.23 [-0.05, 0.49] | 0.04 [-0.24, 0.32] |
| BOLD Signal: GLM t-stat Correlation | -0.05 [-0.35, 0.27] | 0.58 [0.33, 0.75] | 0.66 [0.43,0.80] |

**Note:** RT stands for Reaction time. For accuracy, values represent Mean ± SD. For correlations, values represent Pearson’s r with 95% Confidence Intervals shown in brackets [ ]. The absolute performance metrics are lower than those in the main text because this analysis used a smaller dataset (N=50) and fewer epochs (300) to enable rapid comparison.

**Description of Results:** To evaluate the robustness of our model against the choice of weighting coefficients ($\lambda_{action}$, $\lambda_{BOLD}$), we trained the model under three different schemes using a representative subset (N=50) for 300 epochs. The results reveal a clear trade-off between the behavioral and neural domains. Imbalanced settings failed to model one of the domains effectively: the action-focused scheme yielded high behavioral accuracy but failed to capture neural dynamics (near-zero BOLD correlation), while the BOLD-focused scheme showed the highest neural similarity but resulted in a collapse of behavioral prediction (negative RT correlations).

In contrast, the balanced setting ($\lambda_{action}$ = $\lambda_{BOLD}$ = 1) successfully captured both behavioral dynamics and neural activity patterns simultaneously. While the Stroop RT correlation in this reduced training configuration exhibited a wider confidence interval including zero, this setting represents the most stable and reasonable compromise for the dual-objective optimization required for a Digital Twin Brain.

**Supplementary Table 9. Robustness of Gradient Maps to SmoothGrad Hyperparameters.**

A. Robustness to Noise Level (with Samples =100)

| Noise Level | 0.05 | 0.1 | 0.2 | 0.3 |
| --- | --- | --- | --- | --- |
| 0.05 | >0.99 | >0.99 | >0.99 | >0.99 |
| 0.1 | >0.99 | >0.99 | >0.99 | >0.99 |
| 0.2 | >0.99 | >0.99 | >0.99 | >0.99 |
| 0.3 | >0.99 | >0.99 | >0.99 | >0.99 |

B. Robustness to Sampling Frequency (with Noise Level=0.1)

| Samples | 50 | 100 | 200 |
| --- | --- | --- | --- |
| 50 | >0.99 | >0.99 | >0.99 |
| 100 | >0.99 | >0.99 | >0.99 |
| 200 | >0.99 | >0.99 | >0.99 |

To assess the sensitivity of our biomarker identification to SmoothGrad parameters, we computed group-averaged gradient maps under varying sampling frequencies and noise levels. The table below shows the Pearson correlation coefficients between the gradient map generated with our chosen parameters (samples=100, noise=0.1) and those generated with other settings. The consistently high correlations (r > 0.99) demonstrate that the identified biomarkers are robust to hyperparameter variations
